# Nephron progenitor cell death elicits a limited compensatory response associated with interstitial expansion in the neonatal kidney

**DOI:** 10.1242/dmm.030544

**Published:** 2018-01-01

**Authors:** Sree Deepthi Muthukrishnan, Sergey Ryzhov, Michele Karolak, Leif Oxburgh

**Affiliations:** 1 Center for Molecular Medicine, Maine Medical Center Research Institute, Scarborough, ME 04074, USA; 2 Graduate School of Biomedical Science and Engineering, University of Maine, Orono, ME 04469, USA

**Keywords:** Nephron progenitor cells, F4/80+ macrophages, Interstitial expansion, Renal hypoplasia

## Abstract

The final nephron number in an adult kidney is regulated by nephron progenitor cell availability and collecting duct branching in the fetal period. Fetal environmental perturbations that cause reductions in cell numbers in these two compartments result in low nephron endowment. Previous work has shown that maternal dietary factors influence nephron progenitor cell availability, with both caloric restriction and protein deprivation leading to reduced cell numbers through apoptosis. In this study, we evaluate the consequences of inducing nephron progenitor cell death on progenitor niche dynamics and on nephron endowment. Depletion of approximately 40% of nephron progenitor cells by expression of diphtheria toxin A at embryonic day 15 in the mouse results in 10-20% nephron reduction in the neonatal period. Analysis of cell numbers within the progenitor cell pool following induction of apoptosis reveals a compensatory response in which surviving progenitor cells increase their proliferation and replenish the niche. The proliferative response is temporally associated with infiltration of macrophages into the nephrogenic zone. Colony stimulating factor 1 (CSF1) has a mitogenic effect on nephron progenitor cells, providing a potential explanation for the compensatory proliferation. However, CSF1 also promotes interstitial cell proliferation, and the compensatory response is associated with interstitial expansion in recovering kidneys which can be pharmacologically inhibited by treatment with clodronate liposomes. Our findings suggest that the fetal kidney employs a macrophage-dependent compensatory regenerative mechanism to respond to acute injury caused by death of nephron progenitor cells, but that this regenerative response is associated with neonatal interstitial expansion.

## Abstract (precis)

**Editor's choice:** Formation of the kidney relies on maintaining progenitor cells throughout development. The authors find that apoptotic loss of nephron progenitor cells provokes compensatory proliferation mediated by trophic factors released by phagocytes.

## INTRODUCTION

Nephron number in human kidneys exhibits a 10-fold variability, from 200,000 to >2.0 million per kidney, and low endowment is associated with chronic kidney disease (Bertram et al., 2011). Nephrogenesis ceases in the 36th week of pregnancy in humans and shortly after birth in mice, and there is no evidence that it can be activated in the adult (Hartman et al., 2007; Little and McMahon, 2012). Key factors that determine nephron number during the fetal period are the growth and branching of the collecting ducts (CDs) and the supply of nephron progenitor cells (NPCs) (Hershkovitz et al., 2007). These cell populations lie adjacent to each other within the nephrogenic zone of the embryonic kidney, and iterative reciprocal interactions between them form the basis for new nephron formation (Costantini and Kopan, 2010). Environmental factors are known to influence their availability in the developing kidney. Maternal intake of vitamin A is required for CD growth (Batourina et al., 2001). Maternal dietary factors also influence NPCs, with both caloric restriction and protein deprivation leading to reduced cell numbers through apoptosis (Hokke et al., 2013; Tafti et al., 2011; Welham et al., 2002). Considering the tremendous variability in human nephron endowment, it seems plausible that many factors in addition to diet, such as drugs and toxins, cause such effects.

In this study, we asked what capacity this organogenetic process has to buffer transient reductions in NPC supply. The least differentiated NPCs can be distinguished by their expression of the transcription factor CITED1, and we therefore utilized the tamoxifen-inducible *Cited1-creER^T2^* driver to temporally induce diphtheria toxin subunit A (DTA) expression (Boyle et al., 2008; Brockschnieder et al., 2004). Our analysis of the resulting phenotype shows that NPC loss is compensated for. Macrophages play a key role in providing trophic factors required for this fetal regenerative response, but the regenerative response is associated with interstitial expansion in the neonatal kidney.

## RESULTS

### Ablation of CITED1+ NPCs using inducible-DTA gene expression

**Fig. 1.**
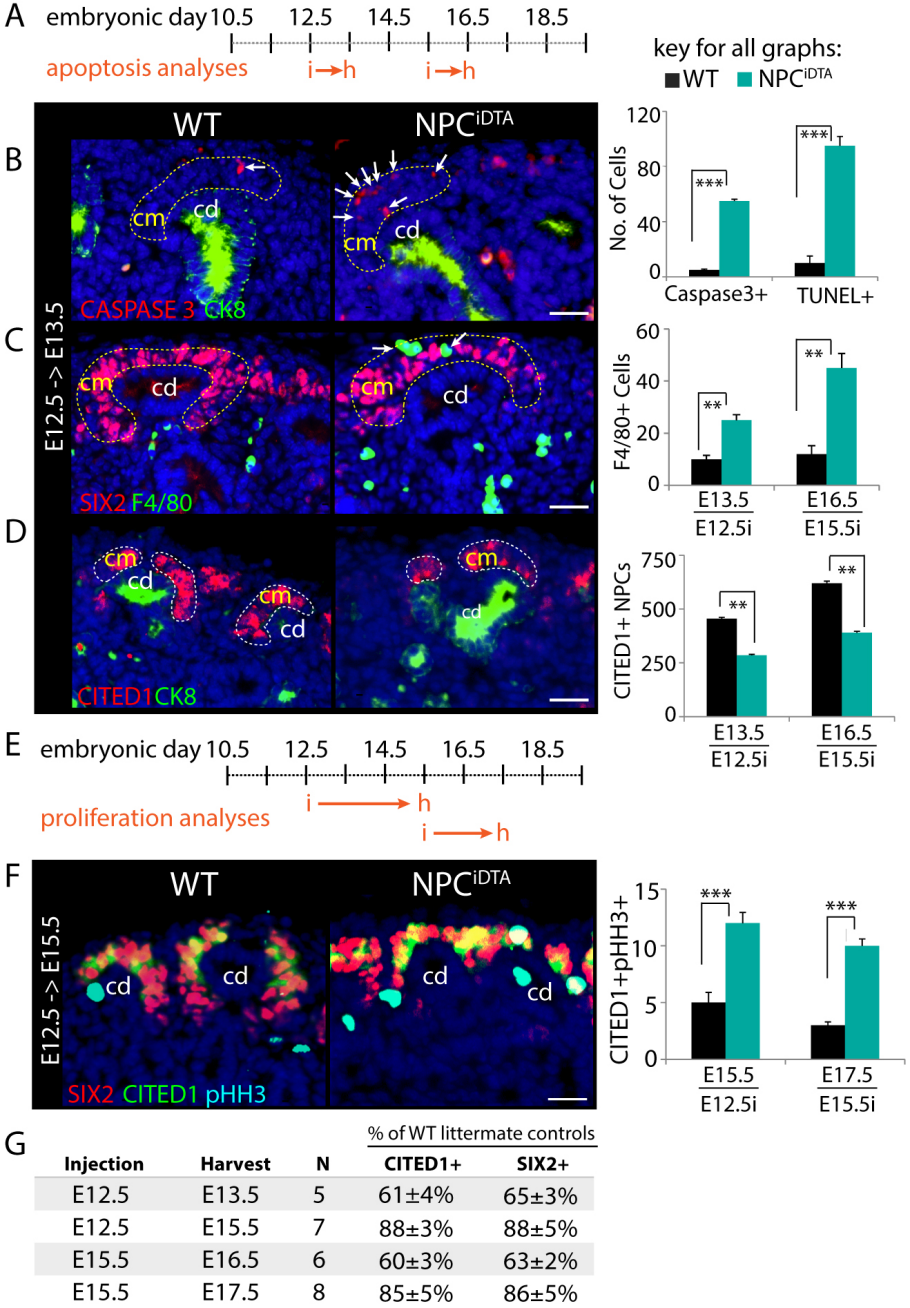
**Transient ablation of CITED1+ NPCs triggers a compensatory increase in proliferation in surviving cells.** (A) Schematic shows the stages at which tamoxifen was injected (i) and kidneys were harvested (h) for apoptosis analyses in WT (*R26RDTA^het^*) and NPC^iDTA^ (*Cited1-creER^T2^*;*R26RDTA^het^*) mice. (B) Caspase3 (red, apoptosis) and cytokeratin 8 (CK8, green, collecting duct) immunostaining in kidneys injected at E12.5. White arrows point to Caspase3+, and F4/80+ cells in the CM (highlighted by dashed lines). Graph shows number of caspase3+ (E13.5) and TUNEL+ (E16.5) cells scored per cap mesenchyme (CM) per kidney section. (C) F4/80 (green, macrophages) and SIX2 (red, CM) staining. Number of F4/80+ cells per CM per kidney section is shown in the graph. (D) CITED1 (red, NPC marker) and CK8 (green, collecting duct) staining. Graph represents the number of CITED1+ NPCs estimated per kidney section. (E) Schematic shows the stages at which tamoxifen was injected (i) and kidneys were harvested (h) for proliferation analyses in WT and NPC^iDTA^ mice. (F) CITED1 (green), SIX2 (red) and pHH3 (cyan blue, proliferation marker) staining. Graphs show quantitation of CITED1+pHH3+ cells per kidney section. (G) Table shows the percentage of CITED1+ and SIX2+ cells remaining after 24 (E12.5→E13.5, E15.5→E16.5), 48 (E15.5→E17.5) and 72 (E12.5→E15.5) hours in NPC^iDTA^ kidneys relative to WT littermates. *N* indicates the number of mice analyzed at each time point. Data represent means±s.e.m. **P<0.005 and ***P<0.005, 2-tailed Student's *t*-test. Scale bars: 100 μm. cd, collecting duct; cm, cap mesenchyme. See also Fig. S1.

Cells expressing the transcription factor CITED1 represent a subset of the SIX2-expressing cap mesenchyme (CM) that is assumed to be the least differentiated NPC based on physical location and evidence that it is refractory to inductive signals (Boyle et al., 2008; Brown et al., 2013; Kobayashi et al., 2008). Cells lose CITED1 expression as they differentiate and this continual loss of cells is balanced by proliferation within the compartment, although studies of NPC motion within the CM indicate that there might also be contribution from cells that have passed out of the CITED1-expressing state (Combes et al., 2016). Cell autonomous factors and signals provided by surrounding cells are essential for maintenance of this equilibrium (Little and McMahon, 2012). To understand whether the nephrogenic niche that maintains this balance is capable of compensating for transient cell loss from the pool, we induced cell death in embryonic day 12.5 (E12.5) or E15.5 CITED1+ NPCs by expressing DTA under the control of the *Cited1-creER^T2^* driver (Boyle et al., 2008; Brockschnieder et al., 2004). A single dose of tamoxifen (3 mg/40 g mouse) was administered to pregnant dams on day 12.5 or 15.5 of gestation and embryos were harvested 24 h after injection (Fig. 1A; Fig. S1A). Cell death was evaluated by activated-caspase3 and TUNEL staining of *Cited1-creER^T2^*;*R26RDTA^het^* (NPC^iDTA^) and *R26RDTA^het^* littermate [wild type (WT)] kidneys. NPC^iDTA^ kidneys induced at both stages displayed a significant increase in caspase3+ cells specifically within the CM compared to WT, which was confirmed by TUNEL staining (Fig. 1B; Fig. S1B). Macrophages are recruited to sites of cell death in the developing mouse embryo and, as expected, we observed a concomitant increase in the number of F4/80+ macrophages surrounding the CM at these time points (Fig. 1C; Fig. S1B) (Camp and Martin, 1996; Hopkinson-Woolley et al., 1994). Cell death in the CM was not elevated at either 48 or 72 h after tamoxifen treatment in NPC^iDTA^ kidneys (Fig. S1C-E). Apoptosis is very rare in the CM of the normal kidney and is typically limited to interstitial cells and differentiating structures undergoing morphogenesis (Foley and Bard, 2002). Activated-caspase3 and F4/80 staining of E16.5 kidneys from untreated NPC^iDTA^ and WT mice confirmed that cell death and macrophage recruitment were specific to tamoxifen-treated NPC^iDTA^ mice (Fig. S1F,G). To confirm NPC depletion within the CM, we performed CITED1 immunostaining. CITED1+ cells were reduced by approximately 40% in CMs from NPC^iDTA^ mice compared to WT (Fig. 1D). Thus, using this inducible cell death system, we achieved specific ablation of CITED1+ NPCs, leaving the majority of the CM intact.

### NPC depletion promotes proliferation of surviving cells

To determine whether surviving NPCs proliferate to compensate for a transient loss of CM cells, NPC^iDTA^ kidneys were co-immunostained for CITED1 and the proliferation marker phospho-Histone-H3 (pHH3). No increase in proliferation of CITED1+ NPCs was seen in NPC^iDTA^ kidneys at 24 h (Fig. S1H-J). Based on this observation, we conducted the proliferation analysis over a longer duration. Mice were either tamoxifen injected at E12.5 and harvested at E15.5, or tamoxifen injected at E15.5 and harvested at E17.5 (Fig. 1E). The number of proliferative CITED1+ cells more than doubled following E12.5 and E15.5 injection, suggesting that there is compensatory proliferation of CITED1+ NPCs in response to cell loss (Fig. 1F). To understand whether this proliferation led to recovery of cell number in the CM, we compared numbers of CITED1+ and SIX2+ cells at 24 h after injection (cell death phase) with cell numbers at 48 or 72 h (proliferative phase). We found that the CITED1+ cell number was 40% lower in NPC^iDTA^ than WT 24 h after tamoxifen treatment both at E12.5 and E15.5, but by 72 h and 48 h after injection, respectively, the difference was only approximately 15% (Fig. 1G). This recovery of cell numbers in the CM is observed using both CITED1 and SIX2 markers, and following injection at either E12.5 or E15.5. Together, these findings suggest that surviving CITED1+ NPCs have the capacity to undergo compensatory proliferation to maintain the niche at different stages of nephrogenesis. To understand whether this compensatory proliferation in the CM associates with proliferation in the CD, we quantified the number of pHH3-expressing cells in cytokeratin 8 stained CD tips. Following tamoxifen injection at E12.5, we see more than a doubling of proliferation after 24 and 48 h (Fig. S1K-M). Following tamoxifen injection at E15.5 we see a modest reduction in CD tip proliferation after 24 h and a small but statistically significant increase of approximately 20% CD tip proliferation after 48 h (Fig. S1N). Thus, compensatory proliferation in the CM is accompanied by proliferation in CD tips.

### E15.5 NPC loss causes cortical thinning and nephron reduction

**Fig. 2.**
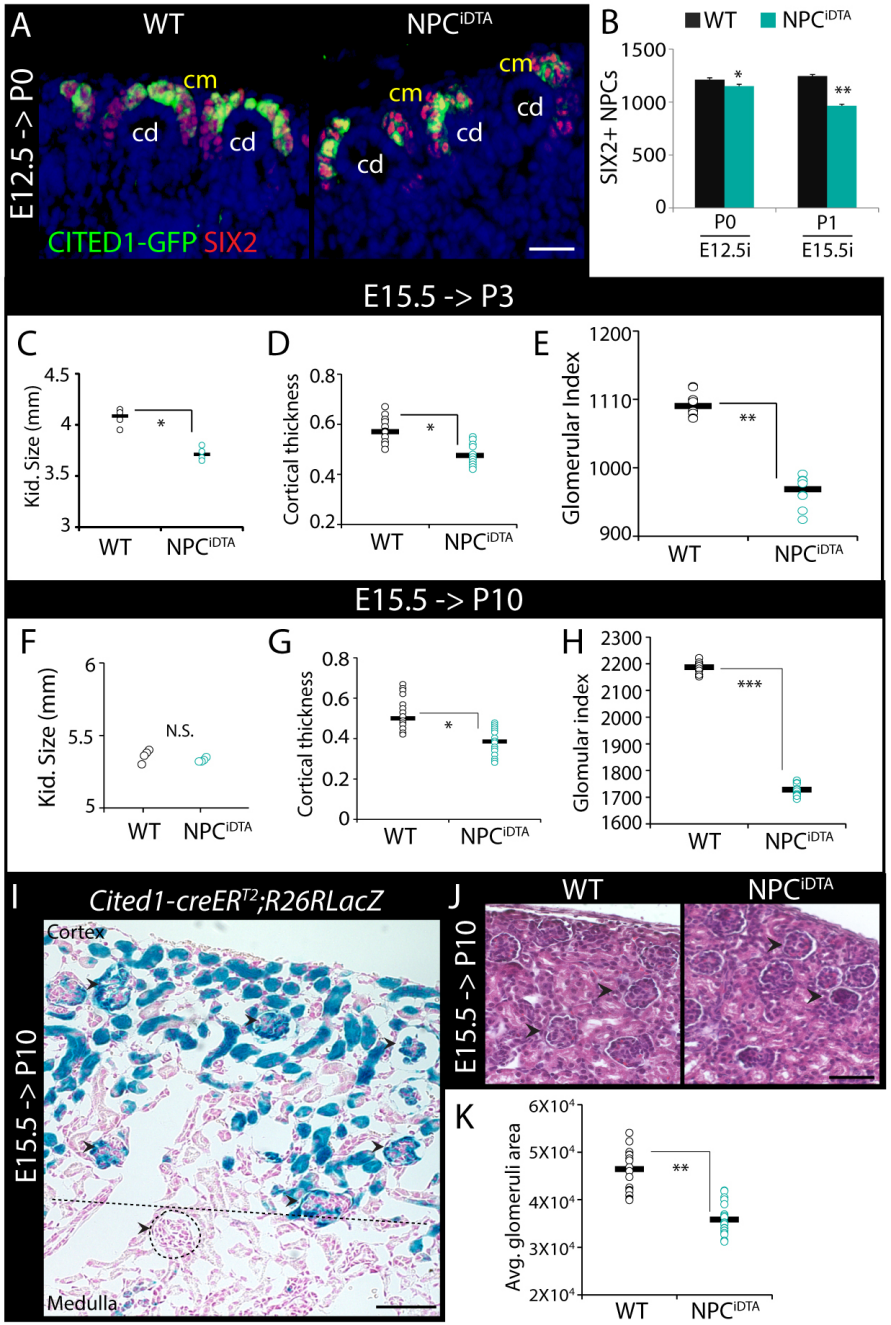
**E15.5 NPC depletion causes reduced nephron number.** (A,B) CITED1-GFP (green) and SIX2 (red) staining in P0 WT and NPC^iDTA^ kidneys (A). (B) The graph shows the number of SIX2+ NPCs scored per section in P0 and P1 kidneys. Number of individual tamoxifen-treated mice analyzed per group (*n*)=5. E12.5i/E15.5i, tamoxifen injection at E12.5/E15.5. (C-H) Kidney size, cortical thickness and glomerular index of P3 and P10 WT and NPC^iDTA^ kidneys. Number of individual tamoxifen-treated mice analyzed per group (*n*)=4. (I) X-gal-stained P10 *Cited1-creER^T2^*;*R26RlacZ* kidneys show that E15.5 CITED1+ NPCs largely contribute to cortical glomeruli (CG) and tubular structures. Nuclei are counterstained with Nuclear Fast Red. Black arrowheads point to X-gal+ glomeruli and dashed circle highlights X-gal– glomeruli localized in the medullary region (below the dashed line). Number of individual tamoxifen-treated mice analyzed per group (*n*)=3. (J,K) H&E-stained sections show CG (black arrowheads) in P10 kidneys (J). (K) Graph shows quantitation of the average area (µm^2^) of CG (*n*=20) measured per kidney section. Data represent means± s.e.m. N.S., not significant (*P*>0.05), *P<0.05, **P<0.005 and ***P<0.005, 2-tailed Student's *t*-test. Scale bars: 150 μm (A), 200 μm (I) and 50 μm (J). cd, collecting duct; cm, cap mesenchyme. See also Fig. S2.

To understand whether NPC^iDTA^ kidneys fully recover from cellular loss, we analyzed kidneys of pups tamoxifen-treated at E12.5 and harvested at postnatal day 0 (P0) and kidneys of pups treated at E15.5 and harvested at P1. NPCs were reduced by approximately 10% in NPC^iDTA^ mice treated at E12.5, suggesting that NPC death at this early developmental stage is relatively efficiently compensated. However, mice treated at E15.5 showed a 24% reduction by P1 (Fig. 2A,B; Fig. S2A). To understand whether this NPC reduction was associated with reduced nephron number, we examined E15.5 tamoxifen-treated WT and NPC^iDTA^ kidneys at P3. P3 NPC^iDTA^ kidneys were significantly reduced in size relative to WT (Fig. 2C). H&E staining revealed reduced cortical thickness and approximately 12% reduction in glomerular number, implying that E15.5 NPC depletion results in reduced nephron number (Fig. 2D,E; Fig. S2B).

To assess whether the organ size reduction was maintained in the juvenile period, we compared WT and NPC^iDTA^ kidneys at P10 following E15.5 tamoxifen induction. Surprisingly, no significant differences were seen in kidney weight or size (Fig. 2F; Fig. S2C-G). The entire nephron is derived from CITED1+ NPCs and, to understand which tubular compartment in the P10 kidney would primarily be affected by tamoxifen induction at E15.5, we conducted a lineage-tracing experiment on *Cited1-creER^T2^*;*R26RlacZ* embryos. β-galactosidase staining of P10 kidneys that had been tamoxifen treated at E15.5 revealed labeling almost exclusively confined to cortical glomeruli (CG) and tubules (Fig. 2I; Fig. S2H). Based on this, we extrapolate that the cells ablated by an E15.5 pulse of tamoxifen in NPC^iDTA^ kidneys are fated to localize strictly to the cortex at P10. The loss of nephron epithelial cells in this region is predicted to result in thinning of the cortex, and indeed we find reduced cortical thickness at P10, and a reduction in glomerular number by 21% relative to WT (Fig. 2G,H; Fig. S2D). In addition to the reduced glomerular number, CG in P10 NPC^iDTA^ kidneys were smaller, and medullary glomeruli (MG), which are not predicted to be affected by E15.5 DTA treatment, were slightly larger compared to WT (Fig. 2J,K; Fig. S2I,J). Immunostaining for the podocyte marker WT1 shows reduced numbers of stained cells in glomeruli compared to WT (Fig. S2K,L). In summary, despite the compensation in organ size seen by P10, ablation of E15.5 CITED1+ NPCs by tamoxifen injection resulted in a reduction of nephron number and glomerular size.

### Expansion of the medullary interstitium in P10 NPC^iDTA^ kidneys

**Fig. 3.**
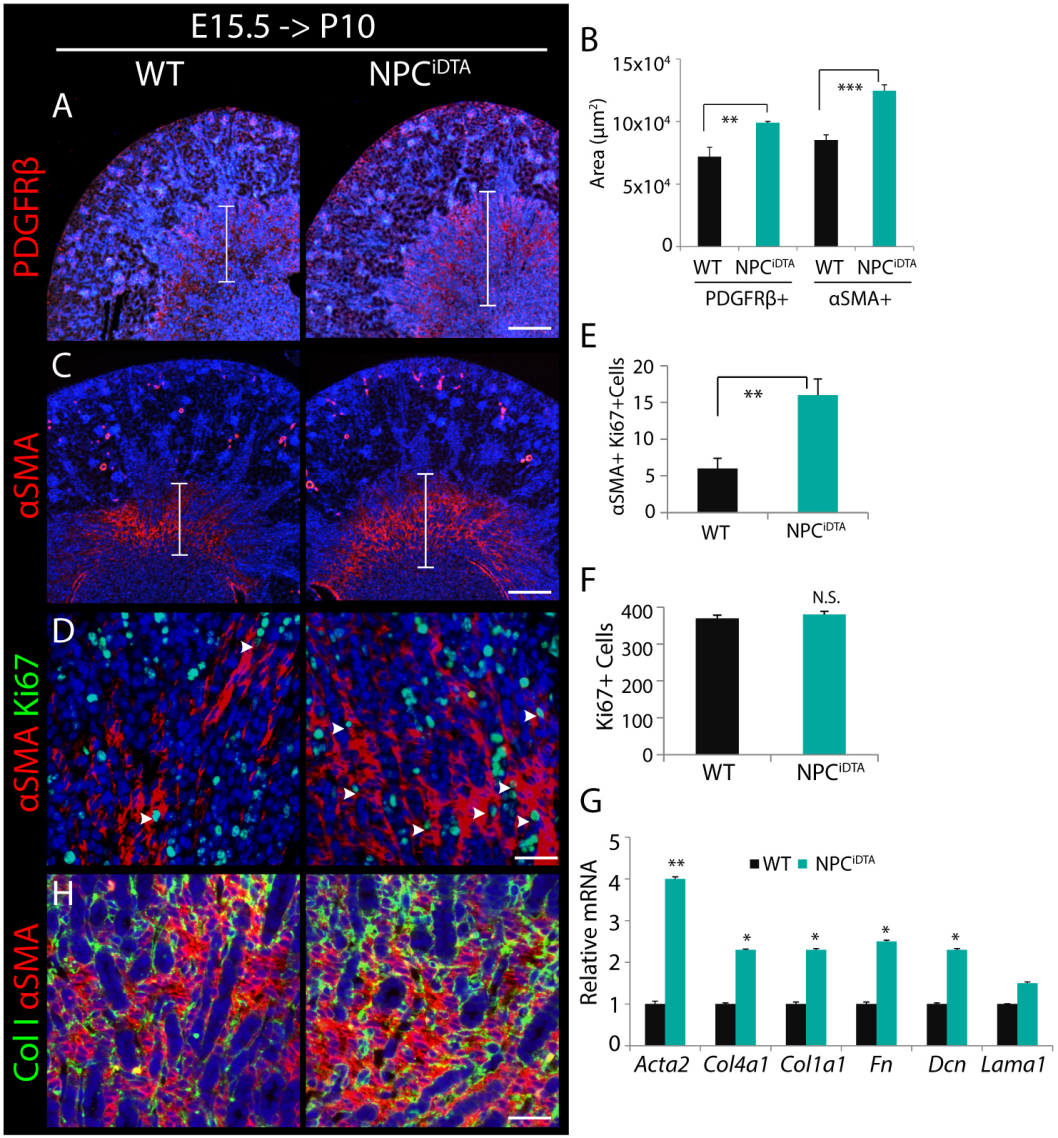
**P10 NPC^iDTA^ kidneys exhibit increased medullary interstitial expansion.** (A-C) PDGFRβ and αSMA (red; medullary interstitium) staining in P10 WT or NPC^iDTA^ kidneys tamoxifen treated at E15.5. Graph shows quantitation of PDGFRβ+ and αSMA+ area in the medullary region per kidney section. The size of the medullary interstitium is indicated by the bracketed area. (D-F) Co-staining of αSMA (red) with Ki67 (green; proliferation marker) in medullary region. Graph shows the number of αSMA+Ki67+ cells (white arrowheads) scored per field (E) and total number of Ki67+ cells per kidney section (F). (G) Transcriptional analysis of *Acta2* and ECM markers (*Col4a1*, *Col1a1*, *Fn*, *Dcn* and *Lama1*) in P10 WT and NPC^iDTA^ kidneys. Four biological replicates (*n*=4) analyzed per genotype. (H) Collagen I (ColI; green) and αSMA (red) co-staining. Data represent means±s.e.m. N.S., not significant (*P*>0.05), *P<0.05, **P<0.005, and ***P<0.0005, 2-tailed Student's *t*-test. Scale bars: 200 μm (A,C), 100 μm (D,H).

An intriguing feature of the P10 kidney in which E15.5 NPCs have been ablated is that it is indistinguishable in size from WT counterparts although nephron number is reduced. Analysis of H&E sections indicated interstitial expansion in the outer medulla (Fig. S2D,E). The pericyte/fibroblast marker PDGFRβ exhibited increased expression in the outer medullary region of NPC^iDTA^ kidneys, and this coincided with increased αSMA expression (Fig. 3A-C) (Humphreys et al., 2010). To understand whether this presumptive pericyte/fibroblast expansion was associated with increased proliferation, we co-stained with the proliferation marker Ki67. Quantitation of αSMA+Ki67+ cells revealed an increase in proliferation of αSMA-expressing medullary interstitium in the NPC^iDTA^ kidneys (Fig. 3D,E). This was specific to the pericyte/fibroblast compartment as overall proliferation of whole NPC^iDTA^ and WT kidneys showed no significant differences (Fig. 3F). The expansion of αSMA-expressing interstitial cells in NPC^iDTA^ kidneys suggests a reactive state similar to scarring. We therefore examined NPC^iDTA^ kidneys for expression of extracellular matrix (ECM) components; collagen IVα1 (*Col4a1*), collagen Iα1 (*Col1a1*), fibronectin (*Fn*) and decorin (*Dcn*) are all elevated in NPC^iDTA^ kidneys (Fig. 3G). Collagen I immunostaining revealed increased deposition in the outer medulla in regions of expanded αSMA (Fig. 3H).

**Fig. 4.**
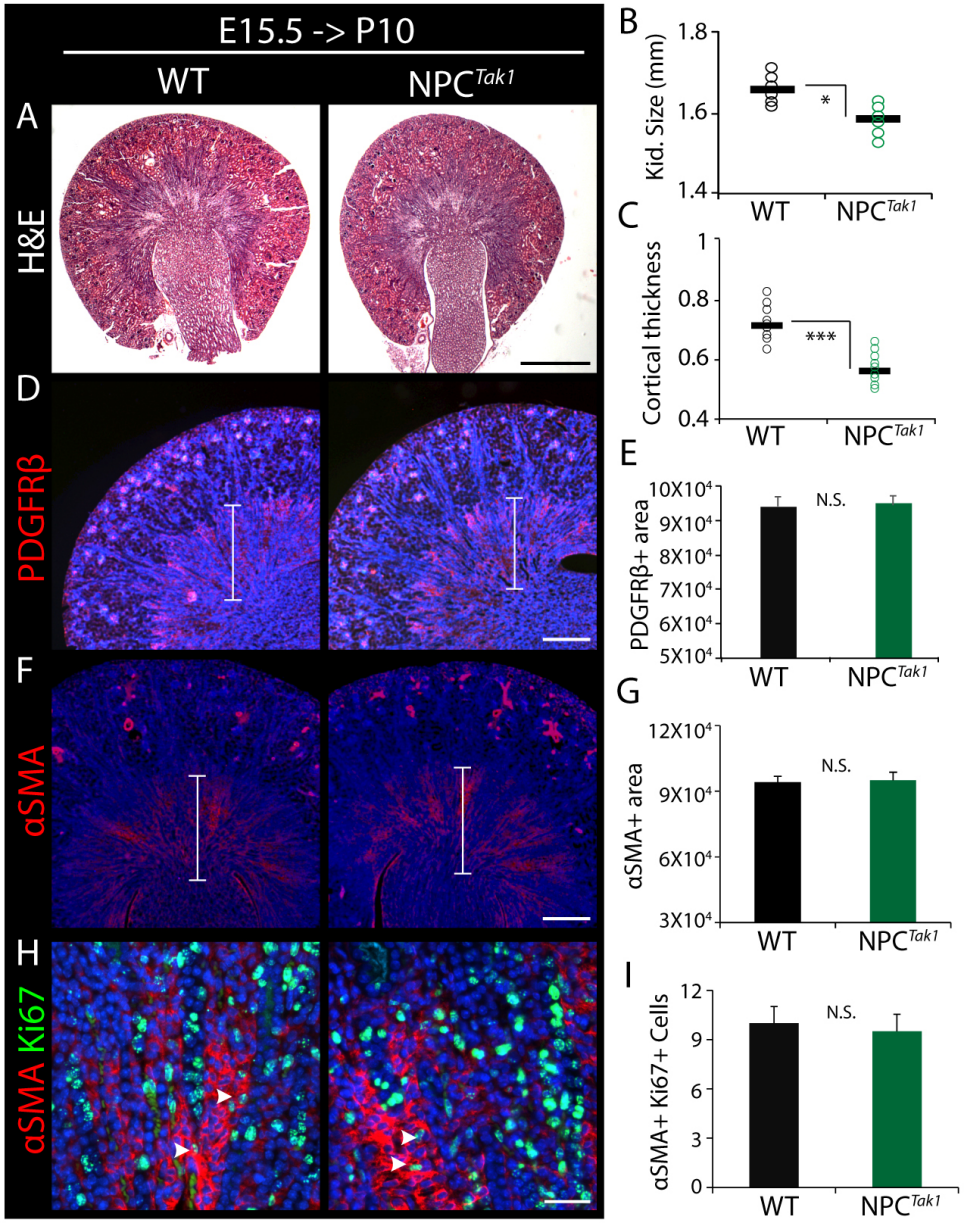
**Interstitial expansion is not associated with impaired proliferation-induced NPC depletion.** (A) H&E-stained images of P10 NPC^Tak1^ (*Cited1-creER^T2^*;*Tak1^c/c^*) and WT littermate controls (*Tak1^c/c^*) tamoxifen treated at E15.5. (B,C) Kidney size and cortical thickness (in mm) of P10 WT and NPC^Tak1^ kidneys. Number of individual tamoxifen-treated mice analyzed per group (*n*)=3. (D-G) PDGFRβ and αSMA (red; medullary interstitium) immunostaining in P10 WT and NPC^iDTA^ kidneys tamoxifen treated at E15.5. Graphs show quantitation of PDGFRβ+ and αSMA+ area (µm^2^) in the medullary region (indicated by brackets) per kidney section. (H,I) Co-staining of αSMA (red) with Ki67 (green; proliferation marker) in the medullary region. Number of αSMA+Ki67+ cells (white arrowheads) was scored per field per kidney section. Data represent means±s.e.m. N.S. not significant (*P*>0.05), *P<0.05, ***P<0.0005, 2-tailed Student's *t*-test. Scale bars: 500 μm (A), 200 μm (D,F) and 100 μm (G). See also Fig. S3.

**Fig. 5.**
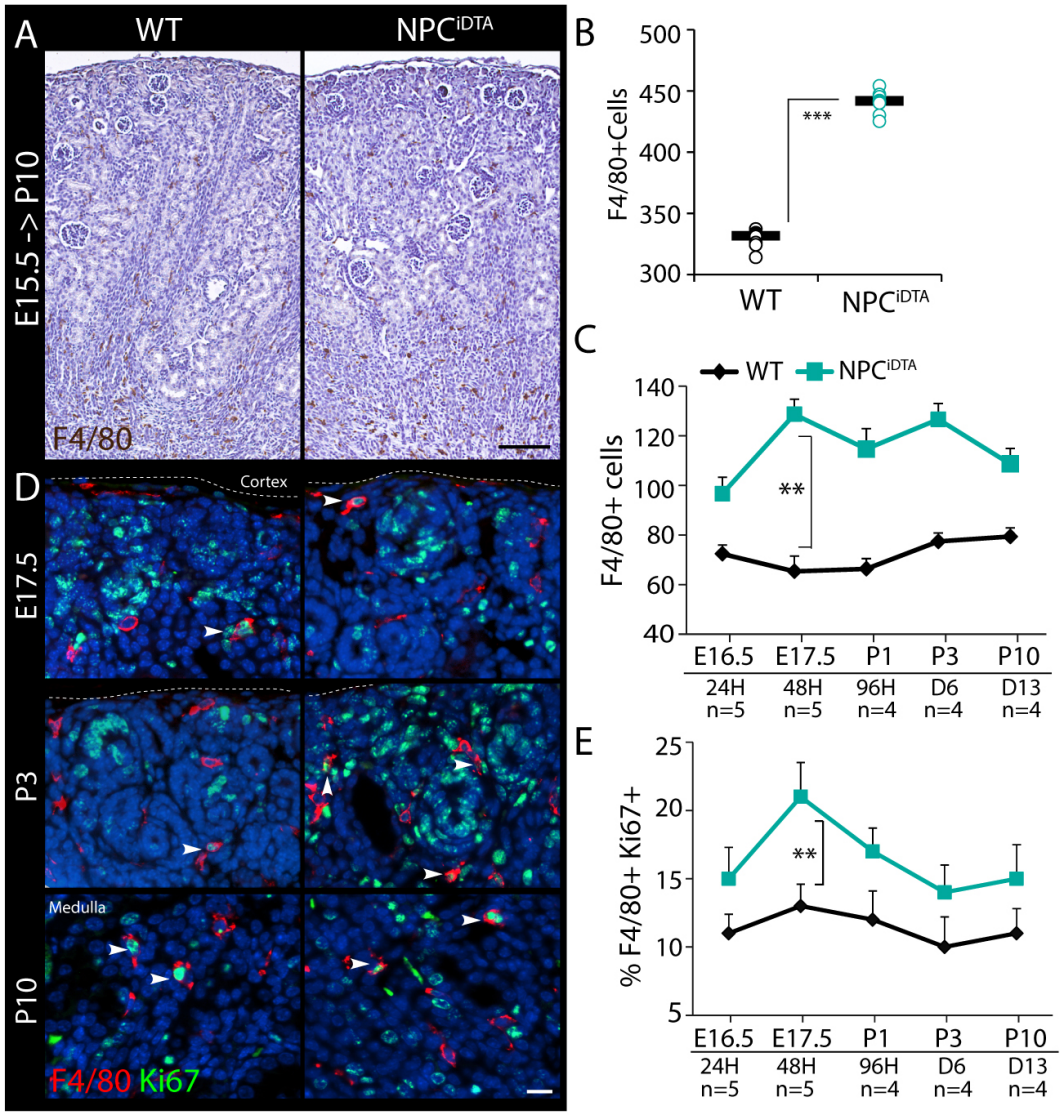
**Increased retention of F4/80+ macrophages in NPC^iDTA^ kidneys.** (A,B) F4/80 (macrophages) staining. Number of F4/80+ cells was scored per kidney section. (C) Number of F4/80+ cells scored at various time points after tamoxifen treatment at E15.5. Cell numbers were normalized to organ size at each time point. (D,E) F4/80 (red) and Ki67 (green; proliferation marker) co-staining. Percentage of F4/80+Ki67+ cells (white arrowheads) per kidney section is shown. *n*, number of individual tamoxifen-treated mice analyzed per group. Data represent means± s.e.m. **P<0.005 and ***P<0.0005, 2-tailed Student's *t*-test. Scale bars: 200 μm (A), 50 μm (D). See also Fig. S3.

To understand whether the interstitial expansion occurred as a consequence of reduced NPC number, we investigated whether a distinct renal hypoplasia model with a comparable transient reduction in NPC number exhibits a similar interstitial phenotype. *Tak1* is an essential component of the proliferation pathway in NPCs, and its inactivation using the *Cited1-creER^T2^* driver results in NPC depletion by approximately 35% in mutants (*Cited1-creER^T2^*;*Tak1^c/c^*) relative to WT littermate controls (*Tak1^c/c^*) after 72 h of tamoxifen treatment (Muthukrishnan et al., 2015). An important feature of this mutant is that cell death is not activated; rather, NPC depletion is caused by failure to proliferate (Fig. S3A-C). NPC^Tak1^ mice were tamoxifen induced at E15.5 and harvested at P10 for comparative analysis with NPC^iDTA^ mice. In contrast to NPC^iDTA^ kidneys, NPC^Tak1^ were smaller than WT littermates at P10 with reduced cortical thickness and glomerular index (Fig. 4A-C; Fig. S3D). PDGFRβ and αSMA staining showed no difference in medullary interstitium distribution or proliferation between NPC^Tak1^ and WT kidneys (Fig. 4D-I). This indicates that the recovery in size of NPC^iDTA^ kidneys through interstitial expansion is a unique response to apoptosis-induced cellular depletion, and is not indirectly caused by loss of NPCs or the pathophysiological changes associated with reduced nephron number. F4/80 staining revealed that macrophage numbers were increased in NPC^iDTA^ kidneys, but not in NPC^Tak1^ kidneys at P10 (Fig. 5A-C; Fig. S3E). Thus, although NPC death induced by E15.5 tamoxifen injection of NPC^iDTA^ is transient, macrophage recruitment is sustained well beyond the pulse of NPC death and indeed also beyond the loss of NPCs at P3 when nephrogenesis terminates. To understand whether macrophage infiltration is indeed sustained, we analyzed F4/80+ cell numbers at 24 h, 48 h, 96 h, 6 days and 13 days after tamoxifen induction at E15.5. F4/80+ cell numbers were normalized to kidney size to account for increased organ growth in WT mice over time and the reduced organ size of E17.5 and P3 NPC^iDTA^ mice. F4/80+ cells showed a significant increase in E17.5 NPC^iDTA^ kidneys and remained high during the postnatal period (Fig. 5E; Fig. S3F). Recent studies indicate that tissue-resident macrophages proliferate locally to maintain their numbers during the embryonic period (Gentek et al., 2014; Sieweke and Allen, 2013). We therefore investigated whether the increase in F4/80+ macrophage numbers at E17.5 is due to increased proliferation. Co-staining with the proliferation marker Ki67 showed the greatest increase in proliferation at E17.5, and only a slight increase in proliferation in the postnatal period (Fig. 5D,E; Fig. S3G). This suggests that the increase in macrophage number at P10 is due to increased proliferation of resident F4/80+ cells immediately after NPC cell death in the embryonic period. To understand whether the increased macrophage number seen at P10 correlates with fibrosis in the adult kidney, we aged animals that had been tamoxifen induced at E15.5 to 8 weeks of age (Fig. S4). Although kidney weight and glomerular number were slightly reduced (Fig. S4A-C), overt scarring was not a feature of the phenotype. Instead, there appeared to be a subtle increase in interstitial collagen I deposition (Fig. S4D,E) with a concomitant increase in *Col1a1*, *Col4a1* and *Fn1* expression (Fig. S4F). Thus, although the kidneys are not overtly fibrotic, they do show increased ECM expression.

### Macrophage CSF1 promotes NPC and interstitial cell proliferation

**Fig. 6.**
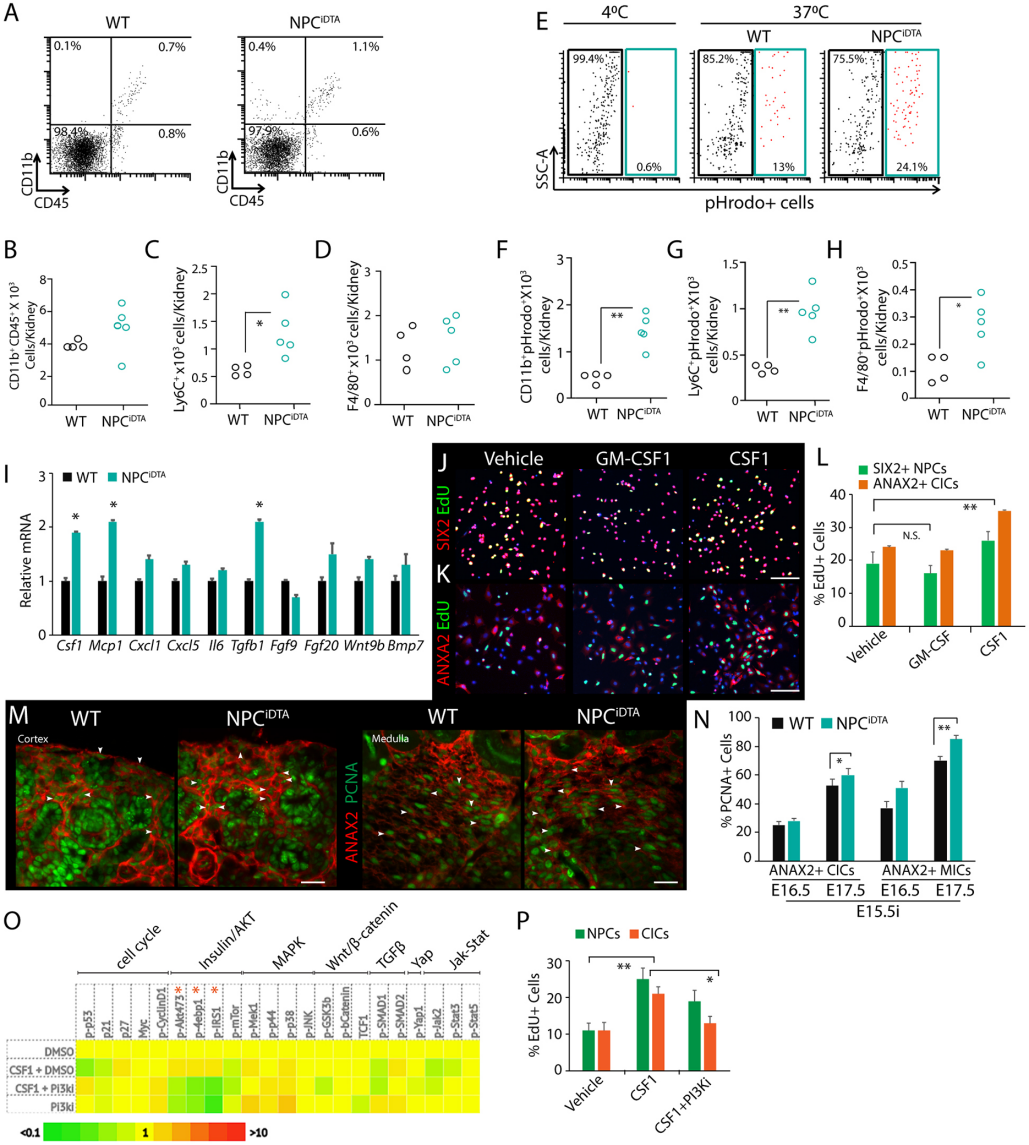
**Functional analysis of macrophages in NPC^iDTA^ kidneys.** (A-D) Representative dot plots show the percentage of CD11b+ and CD45+ cells in 24 h tamoxifen-treated WT (*n*=4) and NPC^iDTA^ (*n*=5) kidneys injected at E15.5. The total cell number was calculated from viable cell counts and expressed as the number of cells per kidney. Numbers of CD11b+CD45+, Ly6C+ and F4/80+ cells were calculated from the corresponding percentage of each cell subpopulation, and total number of cells. (E-H) Dot plot shows percentage of active phagocytes (pHrodo+; blue gate) within the CD11b+CD45+Ly6G− cell population. Data represent means±s.e.m. from 4 experiments. *P<0.05 and **P<0.005, unpaired Student's *t*-test. (I) Transcriptional analysis of chemokines and growth factors in 24 h tamoxifen-treated WT and NPC^iDTA^ kidneys injected at E15.5. Three biological replicates (*n*=3) analyzed per group. (J-L) Proliferation analysis with EdU labeling (green; proliferation marker) of purified E17.5 NPCs (SIX2; red) and CICs (ANAX2; red) treated with vehicle, CSF1 and GM-CSF for 24 h. Graph shows percentage of EdU+SIX2+ NPCs and EdU+ANAX2+ CICs in each condition. Data represent means±s.e.m. from 3 experiments. (M,N) ANAX2 (red; interstitial cells) and PCNA (green; proliferation marker) co-staining. Quantitation of the number of ANAX2+ PCNA+ cells (white arrowheads) in the cortical nephrogenic zone and medullary interstitium. (O) Heat map shows expression profile of various signaling pathway components in NPCs treated with vehicle (DMSO), CSF1 and PI3K inhibitors (PI3Ki). Two biological replicates analyzed per condition. (P) Graph shows percentage of EdU+ NPCs and CICs treated with vehicle (DMSO), CSF1 and PI3Ki. Data represent means±s.e.m. from 2 experiments. *P<0.05 and **P<0.005, 2-tailed Student's *t*-test. Scale bars: 200 μm (J), 50 μm (M). See also Fig. S5.

To better understand the macrophage-mediated injury response in embryonic kidneys, we studied their functional characteristics in E16.5 NPC^iDTA^ tissue immediately after NPC death. Myeloid cells, including monocytes, macrophages and neutrophils, can be distinguished by their expression of CD11b and CD45 markers (Yang et al., 2014). We therefore analyzed single-cell suspensions of whole NPC^iDTA^ kidneys by flow cytometry to understand whether DTA-induced NPC death leads to increased myeloid cell infiltration. Surprisingly, there were no significant differences in proportions of CD45+ and CD11b+CD45+ myeloid cells between NPC^iDTA^ and WT kidneys (Fig. 6A,B; Fig. S5A). There was also no evidence of neutrophil (Ly6G+CD11b+CD45+) infiltration in E16.5 NPC^iDTA^ kidneys, a characteristic of an early myeloid immune response in adult kidney tissue injury (Fig. S5B-D). We did, however, observe a selective increase in Ly6C+ monocytes in NPC^iDTA^ kidneys (Fig. 6C). Ly6C is expressed on monocytes that are recruited to sites of tissue damage, and mature into Ly6C− F4/80+ macrophages in response to phagocytosis and chemokines in the milieu (Rose et al., 2012). Numbers of cells expressing the macrophage marker F4/80+ were not significantly altered between E16.5 WT and NPC^iDTA^ kidneys, consistent with our finding that F4/80+ macrophage number does not show a significant increase until 48 h after tamoxifen injection (Fig. 6D). Taken together, these data indicate that Ly6C+ monocyte recruitment and redistribution of F4/80+ macrophages within the organ is the early response to NPC death in the embryonic kidney. We next assessed the phagocytic capacity of isolated myeloid mononuclear cells (CD11b+Ly6G−) from WT and NPC^iDTA^ kidneys 24 h after tamoxifen induction at E15.5. Ly6C+ monocytes from NPC^iDTA^ kidneys showed increased phagocytic capacity, as did F4/80+ cells (Fig. 6E-H). This suggests that both trafficking monocytes and tissue macrophages phagocytize apoptotic cells in NPC^iDTA^ kidneys.

Next, we assessed NPC^iDTA^ tissue for chemokines expressed by monocytes/macrophages (*Csf1*, *Mcp1*), neutrophils (*Cxcl1*, *Cxcl5*) and inflammatory cytokines (*Il6*, *Tgfb1*) (Duque and Descoteaux, 2014). Macrophages produce a variety of growth factors and therefore we also assayed for changes in tissue expression of factors that are known to promote proliferation in NPCs: *Fgf9*, *Fgf20*, *Wnt9b* and *Bmp7*. NPC^iDTA^ tissue showed increased expression of only monocyte/macrophage chemoattractants *Csf1* and *Mcp1* and not neutrophil chemokines at E17.5, supporting our findings from flow-cytometric analysis, and showed no change in expression of growth factors known to promote proliferation of NPCs (Fig. 6I). We also observed an increase in *Tgfb1*, which promotes fibroblast proliferation in adult kidneys (Fig. 6I) (Blobe et al., 2000; Stahl and Felsen, 2001). This analysis indicates that the chemokines expressed by activated monocyte/macrophages following NPC death may initiate the compensatory response in NPC^iDTA^ kidneys. A trophic role has been reported for CSF1 on CD growth and, to determine whether macrophage chemokines also alter proliferation of other cells in the developing kidney, we tested the effect of CSF1 on nephrogenic zone cells (Rae et al., 2007). Stimulation of purified E17.5 NPCs with CSF1 promoted proliferation compared to vehicle or the closely related chemokine GM-CSF, indicating that proliferation of surviving NPCs after clearance of dead cells by macrophages can be triggered specifically by CSF1 (Fig. 6J-L). We observed a similar effect of CSF1 on interstitial cell proliferation, indicating that CSF1 acts as a mitogen for all 3 major compartments of the nephrogenic zone (Fig. 6J-L). Both cortical (CIC) and medullary interstitial cells (MICs) exhibited increased PCNA expression in E17.5 NPC^iDTA^ kidney tissue, demonstrating that interstitial cells indeed expand in response to NPC death and macrophage recruitment (Fig. 6M,N; Fig. S4E). CSF1 signals via CSF1R, a tyrosine kinase receptor that activates the PI3K pathway, which we have previously shown promotes NPC maintenance (Brown et al., 2011; Mouchemore and Pixley, 2012). To determine which pathway CSF1 may be using to promote proliferation in NPCs, we evaluated changes in the phosphorylation states of 70 distinct signal transduction intermediates using immuno-paired detection (Fig. S5F). At 30 min after stimulation of cells with CSF1, we found a strong profile of PI3K-AKT-insulin signaling (Fig. 6O, Fig. S5G). To determine whether PI3K signaling was responsible for the proliferative effect of CSF1, we treated NPCs with CSF1 in the presence or absence of a PI3K inhibitor and measured the number of proliferating cells (Fig. 6P). We found that inhibition of PI3K indeed does reduce the proliferative response to CSF1, indicating that this pathway is required for CSF1 activation of proliferation in the nephrogenic zone.

### Macrophage depletion reverses interstitial expansion

**Fig. 7.**
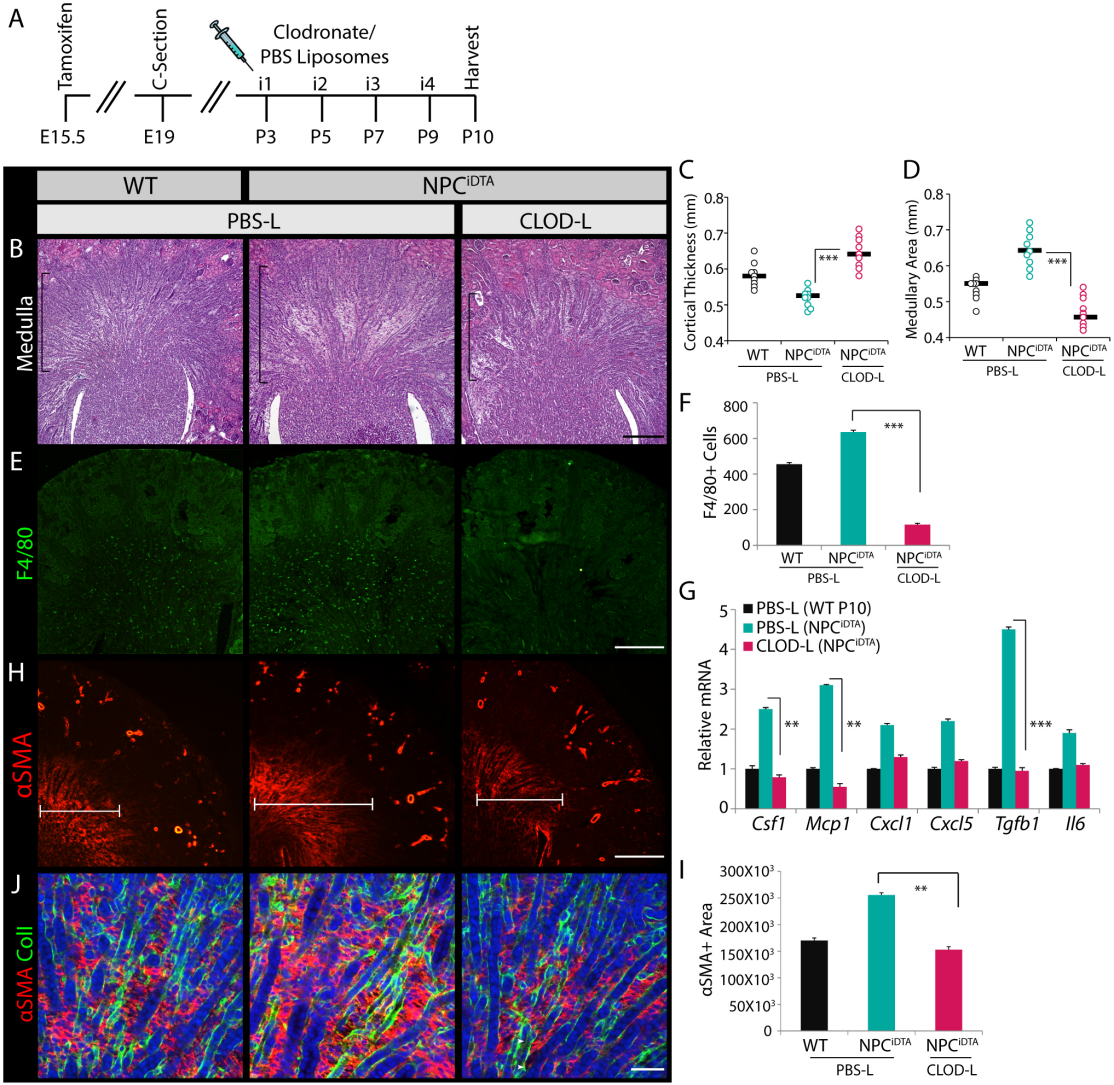
**Postnatal ablation of macrophages resolves interstitial expansion in P10 NPC^iDTA^ kidneys.** (A) Schematic illustrates the timeline of PBS (PBS-L) and clodronate liposome (CLOD-L) administration in WT and NPC^iDTA^ mice. (B-D) H&E-stained sections show the medullary region (bracketed area) of PBS-L- and CLOD-L-treated P10 WT and NPC^iDTA^ kidneys. Cortical thickness (C) and medullary area (D) were measured per kidney section. Number of mice analyzed per group (*n*)=4. (E,F) F4/80 (green) staining. Graph shows the number of F4/80+ cells per kidney section. (G) Transcriptional analysis of chemokines in P10 WT and NPC^iDTA^ kidneys. (H,I) αSMA (red) staining and quantitation of αSMA+ medullary area (µm^2^) per kidney section. (J) Co-staining of αSMA (red) with collagen I (green). Data represent means±s.e.m. **P<0.005 and ***P<0.0005, 2-tailed Student's *t*-test. Scale bars: 200 μm (B,E,H), 100 μm (J). See also Fig. S6.

The implication of our finding that CSF1 promotes both NPC and interstitial cell proliferation is that macrophage-promoted expansion of these cell populations may be synchronized up to P3 when NPCs are depleted, but after this point interstitial cells alone will proliferate. To test this hypothesis, we depleted F4/80+ macrophages after cessation of nephrogenesis to determine whether they are the major cellular source of CSF1 in the kidney and to see if interstitial expansion is resolved in NPC^iDTA^ mice. Macrophages were chemically ablated using clodronate liposomes (Aurora et al., 2014; Van Rooijen and Hendrikx, 2010; Van Rooijen and Sanders, 1994). PBS control (PBS-L) and clodronate (CLOD-L) liposomes were administered to WT and NPC^iDTA^ pups from P3 to P9, and kidney, spleen and liver tissues were harvested at P10 (Fig. 7A). CLOD-L-treated P10 NPC^iDTA^ pups were slightly smaller than PBS-L-treated pups, but the overall difference in body size was not statistically significant (Fig. S6A). Spleen and liver of CLOD-L-treated NPC^iDTA^ pups showed a reduction in F4/80+ cells, confirming global ablation of tissue macrophages (Fig. S6B,C). Morphologically, kidney weights of CLOD-L-treated NPC^iDTA^ pups were markedly reduced compared to PBS-L-treated NPC^iDTA^ pups (Fig. S6D). We observed an increase in cortical thickness and a concomitant reduction in medullary area and interstitial space in CLOD-L-treated NPC^iDTA^ mice (Fig. 7B-D). F4/80 staining confirmed the reduction in macrophage numbers in CLOD-L-treated NPC^iDTA^ kidneys (Fig. 7E,F). Transcriptional analysis of *Csf1* confirmed that F4/80+ macrophages are the major *Csf1*-expressing cells in the kidney. Other macrophage chemokines such as *Mcp1* and *Tgfb1* also showed a marked reduction in CLOD-L-treated NPC^iDTA^ kidneys, indicating that increased expression of chemokine and profibrotic cytokines in the NPC^iDTA^ tissue is caused by resident macrophages (Fig. 7G). Evaluation of the medullary region with interstitial markers PDGFR-β and αSMA showed a significant reduction in their expression in CLOD-L-treated NPC^iDTA^ kidneys (Fig. 7H,I; Fig. S6E). Reverse transcription quantitative real-time PCR (RT-qPCR) analysis also showed a significant decrease in *αSma* expression and ECM proteins (*Col4a1*, *Col1a1*, *Fn* and *Dcn*), and co-staining of COLI with αSMA showed reduced colocalization in CLOD-L-treated NPC^iDTA^ kidneys, indicating that the medullary interstitium is no longer reactive (Fig. 7J; Fig. S6F). Together, these results show that retention of F4/80+ macrophages in the postnatal period after termination of nephrogenesis results in fibrosis in NPC^iDTA^ kidneys.

## DISCUSSION

We propose that F4/80+ macrophages in the fetal kidney not only clear apoptotic cells in the nephrogenic zone, but also provide trophic signals to maintain the NPC population after cell death. Macrophage CSF1 acts as a mitogen through PI3K signaling in the nephrogenic zone, promoting replenishment of both the NPC and interstitial niches (this study) as well as the CD (Rae et al., 2007). However, interstitial cells appear to have a proliferative advantage and expand disproportionately during the compensatory response to NPC apoptosis, resulting in a neonatal kidney with features of fibrosis.

The effects of NPC ablation in our model contrast significantly with those reported in a study employing the same inducible DTA strain to ablate 40% of NPCs at E12.5 using tamoxifen-activated *Gdnf-creER^T2^* (Cebrian et al., 2014). Despite an almost identical degree of NPC ablation at the same developmental time, NPCs in the previous study did not display a compensatory increase in proliferation in *Gdnf-creER^T2^*;*DTA* kidneys. In this model, both CD growth and branching were reduced, and the postnatal *Gdnf-creER^T2^*;*DTA* kidney was severely hypomorphic. The most obvious difference between these experiments is the domain of cells in which apoptosis is induced. *Gdnf* is expressed throughout the CM, whereas *Cited1* is expressed in only a subset of these cells. Thus, a larger number of cells is lost following ablation of 40% of *Gdnf*-expressing cells compared to ablation of 40% of *Cited1*-expressing cells and it is possible that there is a threshold for NPC loss within the CM, above which compensation is not possible.

A different and intriguing possibility is that macrophages may be lost in the *Gdnf-creER^T2^*;*DTA* kidney, preventing the compensatory effects that we propose. The authors observe a population of cells in the interstitium that is lineage-tagged by *Gdnf-creER^T2^* that they speculate may be fibroblasts, macrophages or astrocytes, and previous work has shown a role for *Gdnf* expression in macrophages (Cebrian et al., 2014; Hashimoto et al., 2005). Single-cell expression analysis demonstrates that *Gdnf* is expressed in a subset of MEIS1-expressing stromal cells in the developing kidney (Magella et al., 2017, in press). The exact identity of these *Gdnf*-expressing cells outside the CM remains uncertain as MEIS1 marks both renal interstitial fibroblasts and the myeloid lineage (Calero-Nieto et al., 2014; Mildner et al., 2013). One interesting possibility is that *Gdnf-*expressing macrophages are lost concomitantly with NPCs upon tamoxifen induction of *Gdnf-creER^T2^*;*DTA* mice, resulting in loss of trophic macrophage signals such as CSF1 to NPCs and attenuation of the compensatory response causing nephron loss that is more severe than that seen in the *Cited1-creER^T2^*;*DTA* mouse, where only cells of the CM are ablated. Another possible explanation for the difference in severity is that *Gdnf*-expressing interstitial fibroblasts outside the cap contribute to the repair response, perhaps by providing GDNF to maintain branching, and therefore loss of these cells in the *Gdnf-creER^T2^*;*DTA* mouse would cause more severe nephron loss.

From our findings, we postulate that the fetal kidney responds to NPC death by engaging a rapid repair system based on pro-nephrogenic properties of macrophages. Situations in which this system may buffer the organogenetic process include malnutrition and protein deprivation. Studies of the *Meox2cre*;*Csf1* conditional knockout mouse in which *Csf1* is inactivated in all cells of the embryo have not identified any perturbations in kidney development, and it therefore appears that utilization of this mechanism is limited to injury situations (Harris et al., 2012). Macrophage reporter mice have revealed that these cells are present during kidney development, mostly distributed around forming nephrons where there is continual low-level apoptotic cell loss due to morphogenetic remodeling (Henson and Hume, 2006; Rae et al., 2007). However, due to the lack of a viable mouse strain that is devoid of macrophages, it is not possible to critically test the hypothesis that macrophages play an essential role in normal nephrogenesis. In the adult, the role of macrophages is ambiguous: although some studies of macrophage ablation have concluded that these cells are injurious following kidney damage, there is also strong evidence that they play a protective role (Duffield et al., 2005; Ferenbach et al., 2012; Henderson et al., 2008). Perhaps this lack of clarity is a reflection of the functional heterogeneity of macrophages and the paucity of strategies to target individual subsets. Our study suggests a complex role for macrophages in the kidney during embryonic development, and we are similarly limited in the tools that we can use to distinguish between the roles of different macrophage subpopulations. We cannot rule out the possibility that a quorum-sensing mechanism within the CM contributes to the compensatory proliferative response. In this scenario, cells of the CM would provide proliferative signals to their neighbors to compensate for a reduction in numbers. However, our expression analysis of *Fgf9*, *Fgf20*, *Wnt9b* and *Bmp7* reveals only minor fluctuations in expression in the proliferative phase following DTA ablation, and does not provide robust support for involvement of the pathways that are known to control CM renewal. Further studies on the effects of nutrition, toxins and genetic deficiencies in conjunction with new technologies for inactivation of distinct macrophage populations will be needed to clarify the contexts in which this injury response is activated.

## MATERIALS AND METHODS

### Mouse strains

Animal care was in accordance with the National Research Council (US) Guide for the Care and Use of Laboratory Animals. Protocols were approved by the Institutional Animal Care and Use Committee of Maine Medical Center. *Cited1-creER^T2^* mice were maintained on an FVB/NJ background. *R26R^EGFP-DTA^*, *Tak1^c/c^* and *R26RlacZ* mice were maintained on a C57BL/6 background (Boyle et al., 2008; Brockschnieder et al., 2004; Liu et al., 2006; Soriano, 1999). Tamoxifen (T5648-1G, Sigma) was administered to pregnant dams intraperitoneally at 3 mg per 40 g mouse. Due to high frequency of miscarriage of tamoxifen-treated pregnant dams, pups were delivered by cesarean section (c-section) at E18.5 and transferred to lactating foster mothers to generate animals for postnatal time points.

### Morphometrics

Kidney weight (in mg) measurements were normalized to body weight (in g) to account for differences in overall body size. For kidney size measurements, images of dissected whole kidneys were taken using a stereomicroscope and the pole-to-pole distance of each kidney was calculated using Spot 5.1 Imaging software. Cortical thickness (in mm) measured as cross-sectional distance from the outer cortex to the outer stripe of the outer medulla and medullary area (distance from outer stripe – inner medulla) was measured on H&E-stained whole kidney images in 5 different regions per kidney. Mean of the cortical thickness and medullary area measurements from 3-5 regions per kidney for each animal were counted.

### Histology, X-gal and immunostaining

Dissected whole kidneys were fixed in 4% paraformaldehyde for 15-30 min (E13.5-E17.5), 1 h (P0-P3) or 2-4 h (P10 and 8 weeks) at room temperature. Thin (5 µm) paraffin-embedded serial sections were generated for immunofluorescence or immunohistochemistry. For X-gal staining, kidney tissue was fixed in X-gal pre-fix solution (37% formaldehyde, 25% glutaraldehyde) for 15-30 min at room temperature. Tissue was embedded in OCT freezing medium (TissueTEK) and 5-µm frozen sections were generated for staining. For immunofluorescence staining, sections were incubated with blocking buffer containing phosphate buffered saline (PBS), 1% bovine serum albumin (BSA), 5% serum of secondary antibody species (Jackson ImmunoResearch) and 0.05% hydrogen peroxide (Sigma). Primary antibodies were diluted in PBS and incubated at 4°C overnight. Antibody information is listed in Table S1. Alexa-Fluor-488/568/647- or streptavidin-conjugated secondary antibodies were used at 1:250 for detection of labeled cells. Nuclei were stained using DAPI (Molecular Probes) for immunofluorescence and hematoxylin or Nuclear Fast Red (NFR; Sigma) for immunohistochemistry. TUNEL staining was performed using the ApopTagPlus Peroxidase *In situ* apoptosis detection kit (Millipore) according to the manufacturer's instructions.

### Estimation of cell number, apoptosis and proliferation index: NPC numbers

CITED1+ NPC and SIX2+ NPC number in WT and NPC^iDTA^ kidneys was estimated manually by scoring the total number of stained cells in 10 serial sections spaced 100 µm apart per kidney.

#### F4/80+ macrophage counts

F4/80+ cells localized in the CM of embryonic kidneys were scored for a minimum of 5 serial sections 100 µm apart per kidney. F4/80+ cell number in whole kidneys from pups was scored in serial sections per kidney. The total cell number was normalized to kidney size to account for reduction/increase in organ size of WT and NPC^iDTA^ kidneys at various time points.

#### Apoptosis index

Apoptosis index was determined by scoring caspase3+ and TUNEL+ cells in the CM per kidney section. A minimum of 5 serial sections 100 µm apart were scored per animal.

#### Proliferation index of CITED1+ NPCs

Proliferation index of CITED1+ NPCs was estimated by scoring NPCs co-stained with pHH3 and CITED1. The average number of CITED1+ pHH3+ cells was scored from 10 serial sections 100 µm apart per kidney.

#### Proliferation of medullary interstitial cells

Proliferation of medullary interstitial cells in P10 kidneys was estimated from αSMA and Ki67 co-stained sections. The average number of αSMA+Ki67+ cells was scored per random field (*n*=10 fields) per kidney per animal. The number of proliferating CICs and MICs in the embryonic kidneys was determined by ANXA2+ (interstitial marker) and PCNA+ (proliferation marker) co-staining. A minimum of 5 serial sections 100 µm apart per kidney per genotype were analyzed and the numbers of ANXA2+PCNA+ cells were averaged. Data represent means±s.d. for each animal per experimental group. The number of tamoxifen-treated animals (*n*) analyzed at each time point is indicated in the respective figures.

### Glomerular index and glomerular area

Whole kidneys from P3 and P10 mice were serially sectioned and stained with H&E. The number of glomeruli was scored every 100 µm. The total number of glomeruli scored from 10 to 15 (1-1.5 mm) sections is depicted as the glomerular index per kidney (Brown et al., 2015). For measuring cortical and medullary glomerular area, images of H&E-stained serial kidney sections were taken using a stereomicroscope at magnification of 40×. CG were defined as glomeruli localized in the cortex extending up to the outer stripe of outer medulla; the remaining glomeruli were scored as MG. An area of 20 CG and 10 MG from random fields in serial sections that are 100 µm apart was calculated for 10 sections per kidney using FIJI/ImageJ software and the average area of glomeruli was calculated (Stojanovic et al., 2012).

### Quantitation of αSMA and PGFRβ area

Images of P10 whole kidney sections stained with αSMA and PDGFRβ were taken at magnification of 5× on a stereomicroscope using Spot 5.1 Imaging software. Images were analyzed by FIJI/ImageJ. PDGFRβ+ and αSMA+ area was marked for each section and measured using the area function, and the mean area per μm^2^ from a minimum of 3 kidneys per genotype was calculated.

### RT-qPCR

RNA extraction from embryonic (E16.5, E17.5) and P10 whole mouse kidneys was performed using RNeasy Minikit (Qiagen). The concentration of RNA was measured using a NanoDrop2000 Spectrophotometer (Thermo Fisher Scientific), and a final concentration of 250 ng/μl of RNA was used for cDNA synthesis by iScript™ Reverse Transcription Super Mix (Bio-Rad). RT-qPCR was performed using iQ-SYBR Green Super mix (Bio-Rad). Primer sequences of genes are listed in Table S2. Fold changes were normalized to the housekeeping gene *Gapdh* and average values (means±s.e.m.) of 3 technical replicates and from a minimum of 3 biological replicates (*n*=3) are shown in the figures. *P*-values were calculated using a 2-tailed Student's *t*-test and *P*<0.05 was considered significant.

### Dissociation of mouse embryonic kidneys for flow cytometry

Dissected kidney tissue was minced and digested with an enzyme mix containing collagenase IV (Life Technologies; 10 mg/ml) and DNase I (Sigma; 100 units/ml) dissolved in DMEM media for 45 min at 37°C. Digested tissue was filtered sequentially through 100 µm and 70 µm nylon filters and a 30 µm pre-separation filter (Miltenyi Biotec) into 50-ml tubes containing DPBS buffer (Ca−, Mg−) and placed on ice for 2 min to kill enzymatic activity. Filtrate containing single cells was centrifuged for 5 min at 500 ***g***, and resuspended in FACS buffer (PBS, 0.5% BSA, 2 mM EDTA). Cells were incubated with TrueStain fcX (BioLegend) reagent to prevent non-specific binding and stained with the following antibodies: PeCy7-conjugated Ly6C (HK1.4), F4/80-APC (BM8), PerCP-conjugated CD45 (30-F11) or Ly6G-PerCP/Cy5.5 (1A8) and APC/Cy7-conjugated CD11b (M1/70) (BioLegend). After incubation for 25 min at 4°C cells were washed and analyzed using the MACSQuant Analyzer 10 (Miltenyi Biotec). The activity of myeloid phagocytes was measured using *E. coli* BioParticles labeled with the pH-sensitive fluorogenic dye pHrodo^TM^ (Thermo Fisher Scientific) according to the manufacturer's protocol. Briefly, approximately 3×10^5^ of pHrodo Red BioParticles were added to the single-cell suspension (10^5^ cells; 3:1 ratio) and incubated at 4°C (control) or 37°C. Phagocytosis was stopped after 1 h incubation; cells were labeled with antibody against cell surface markers and analyzed by flow cytometry. Dead cells and pHrodo BioParticles, not phagocytized by myeloid cells, were excluded from analysis using DAPI. Active phagocytes were defined as pHrodo+CD11b+CD45+ cells. The pHrodo+ gate was established using cell suspension incubated with pHrodo BioParticles at 4°C (no phagocytosis).

### Proliferation analysis of isolated NPCs and CICs

Total nephrogenic zone cells were isolated from E17.5 Institute of Cancer Research (ICR) mice by enzymatic digestion as previously described (Brown et al., 2011). Enrichment for CITED1+ cells (referred to as NPCs) and purification was performed by negative depletion with magnetic activated cell sorting (MACS) using phycoerythrin (PE)-conjugated antibodies and anti-PE microbeads according to the manufacturer's protocol (Miltenyi Biotec) (Brown et al., 2013). The positive fraction from the MACS sorting was used to obtain the CICs. Purified NPCs and the mixed CICs were cultured in monolayer in Keratinocyte Serum Free Media (KSFM; Thermo Fisher Scientific) supplemented with 50 ng/ml rh-FGF2 (R&D Systems) and 100 U/ml penicillin-streptomycin. The identity of purified NPCs and mixed culture CICs were analyzed using anti-SIX2 for NPCs and anti-ANNEXINA2 (ANXA2) for CICs.

For proliferation analysis, NPCs and CIC cultures were treated with vehicle, mouse CSF1 or GM-CSF (10 ng/ml; BioLegend) for 24 h. For small-molecule-inhibitor experiments, NPC and CIC cultures were pretreated with small-molecule inhibitors to 2.5 µM ERK (ERK activation inhibitor peptide II, 328005; EMD Millipore) and 50 µM PI3K (LY294002, TOCRIS). Cultures were incubated with 20 μM EdU (Click-iT^®^ EdU Alexa Fluor^®^ 488 Imaging Kit; Life Technologies) 4 h after growth factor stimulation and pulse-chased for 20 h. Fixation, permeabilization and click-iT reaction was performed according to the manufacturer's instructions. Alexa-Fluor-568 secondary antibody was used to visualize the SIX2 and ANXA2 staining. Nuclei were stained with Hoechst 33342 (Life Technologies). A total of 5-10 images were taken per well for each condition with a minimum of 3 biological replicates and from 3 independent experiments. Pooled images were analyzed by FIJI/Image-J and the number of EdU+SIX2+ NPCs or EdU+ANXA2+ CICs were scored and divided by the total number of SIX2+ or ANXA2+ cells to determine the percentage of proliferating NPCs and CICs.

### ActivSignal pathway analysis

NPCs were purified as previously described and plated in serum-free medium (Brown et al., 2015; Muthukrishnan et al., 2015). For inhibitor treatment conditions, cells were pretreated for 60 min with PI3K inhibitor (Tocris; LY294002). CSF1 (10 ng/ml; BioLegend) or vehicle control (DMSO) was then added and cells were fixed using 4% paraformaldehyde. Cells were then subjected to immune-paired antibody detection as described on https://www.activsignal.com/service/. Briefly, for each of the 70 signaling intermediates measured, two separate antibodies were bound to the cells. These antibodies are tagged with oligonucleotide motifs allowing a unique primer set to be used to detect co-binding of antibodies by qPCR.

### Clodronate liposome administration in mice

Clodronate and PBS control liposomes were procured from clodronateliposomes.org (Van Rooijen and Hendrikx, 2010). At E18.5, pups were c-sectioned from E15.5 tamoxifen-treated pregnant dams and transferred to foster mothers. Clodronate or PBS liposomes were administered to pups retro-orbitally from P3 to P9 at a concentration of 10 µl per 1 g body weight on alternate days. Kidneys, liver and spleen were harvested at P10.

### Statistical analyses

A 2-tailed Student's *t*-test was performed to determine the statistical significance, and *P*-values less than 0.05 were deemed significant.

## Supplementary Material

Supplementary information
