# Supplementary material for: Nephron progenitor cell death elicits a limited compensatory response associated with interstitial expansion in the neonatal kidney
Source: Dis Model Mech. 2018 Jan 1;11(1):dmm030544. doi: 10.1242/dmm.030544 (PMC5818074; doi:10.1242/dmm.030544)
Supplement: Supplementary information [file dmm-11-030544-s1.pdf]

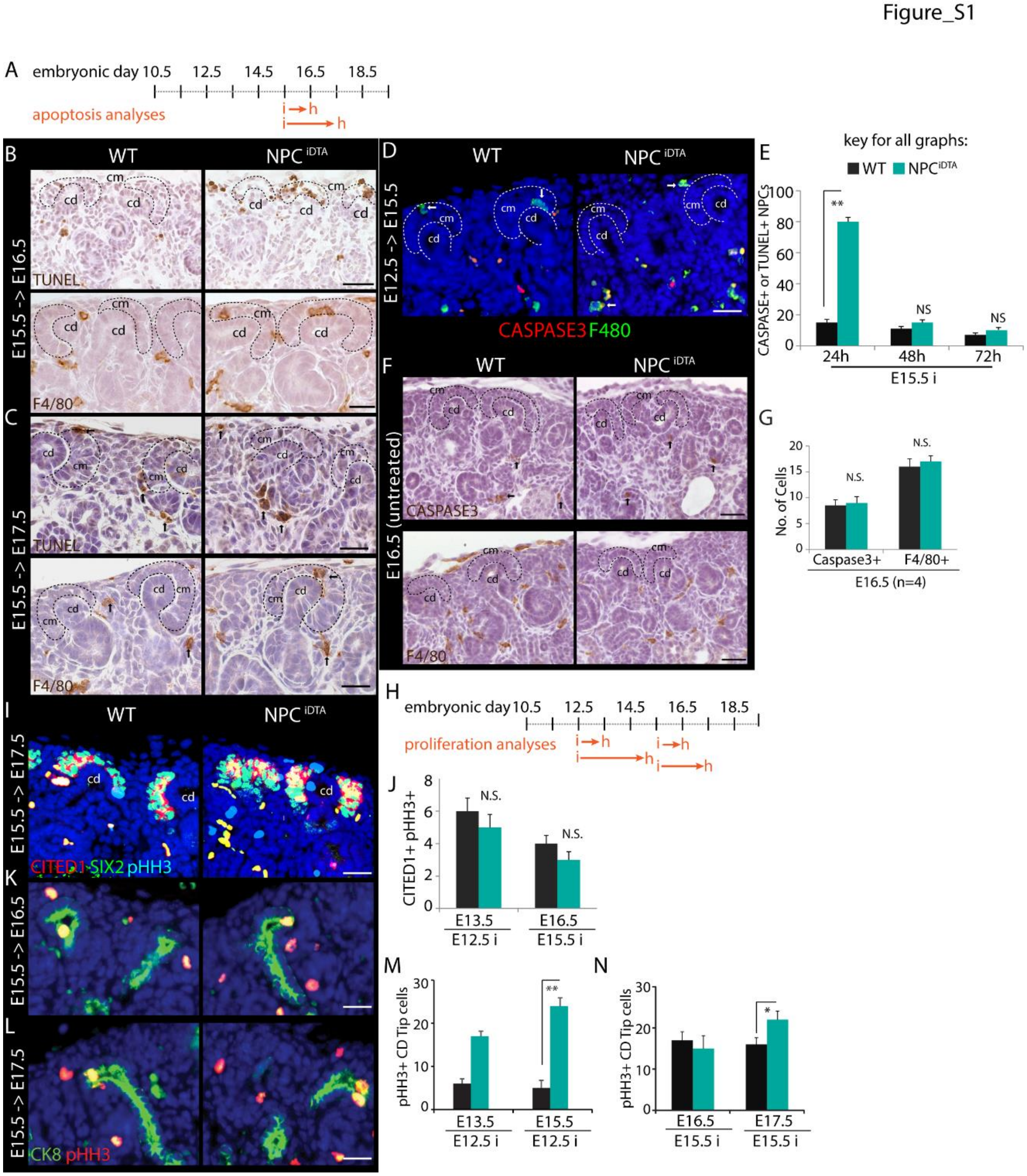

Figure S1: Apoptosis and proliferation analyses in NPC^{iDTA} kidneys

(A) Schematic shows the stages at which tamoxifen was injected (i) and kidneys were harvested (h) for apoptosis analyses in WT (*R26RDTA^{het}*) and NPC^{iDTA} (*Cited1-creER^{T2}*;*R26RDTA^{het}*) mice.

Disease Models & Mechanisms • Supplementary information

(B,C) TUNEL (apoptosis) and F4/80 (macrophage) staining.

(D-G) CASPASE 3 (apoptosis) and F4/80 staining. Graphs show number of Caspase3+ and F4/80+ cells (black, white arrows) in the cap mesenchyme per kidney section. Number of mice (n=4) analyzed per group in (G).

(H) Schematic shows the stages at which tamoxifen was injected (i) and kidneys were harvested (h) for proliferation analyses in WT and NPC^{iDTA} mice.

(I,J) CITED1 (red), SIX2 (green) and pHH3 (cyan blue, proliferation) staining. Number of CITED1+ pHH3+ scored per kidney 24 hours after injection at E12.5 and E15.5.

(K-N) Cytokeratin 8 (CK8, green) and pHH3 (red) staining. Graphs show number of pHH3+ CK8+ CD tip cells per kidney. Data represents mean ± SD. N.S. not-significant (P>0.5), * P<0.05 and ** P<0.005, Student's t-test. Scale bars, 100 μm in all images. Abbreviations: cd-collecting duct; cm-cap mesenchyme.

Disease Models & Mechanisms • Supplementary information

Figure_S2

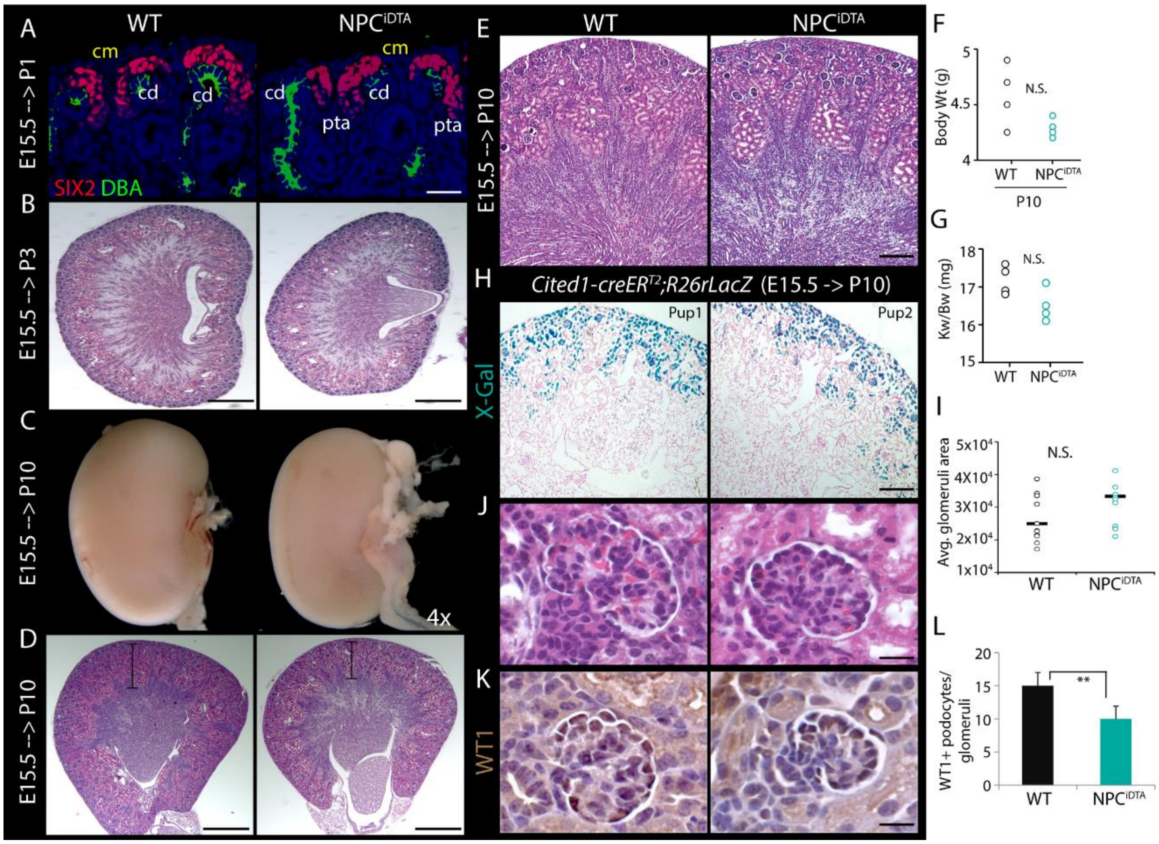

Figure S2: Effects of E15.5 NPC depletion in P3 and P10 NPC^{iDTA} kidneys

(A) SIX2 (red) and DBA lectin (green, collecting duct) staining in P1 WT and NPC^{iDTA} kidneys injected at E15.5.

(B) H&E stained images of P3 WT and NPC^{iDTA} kidneys injected at E15.5.

(C-E) Whole mount and H&E stained images of P10 WT and NPC^{iDTA} kidneys injected at E15.5.

(F,G) Body weight and kidney weight of P10 WT and NPC^{iDTA} kidneys.

(H) X-gal stained P10 *Cited1-creER*^{T2};*R26RLacZ* kidneys show contribution of E15.5 CITED1+ NPCs to largely cortical glomeruli and tubular structures. Nuclei are counterstained with nuclear fast red.

(I) Area of medullary glomeruli (μm2, MG, n=10) measured in P10 WT and NPC^{iDTA} kidneys.

(J-L) H&E and WT1 (brown, podocyte) staining in glomeruli. Number of WT1+ podocyte number per glomeruli is indicated in the graph. 25 glomeruli (G) per group were analyzed.

Data represent mean ± SD. N.S. not-significant (*P*>0.05), ***P*<0.005, Student's t-test. Scale bars, 100 μm (A), 500 μm (D) and 50 μm (I,J). Abbreviations: cd-collecting duct; cm-cap mesenchyme, pta - pre-tubular aggregate.

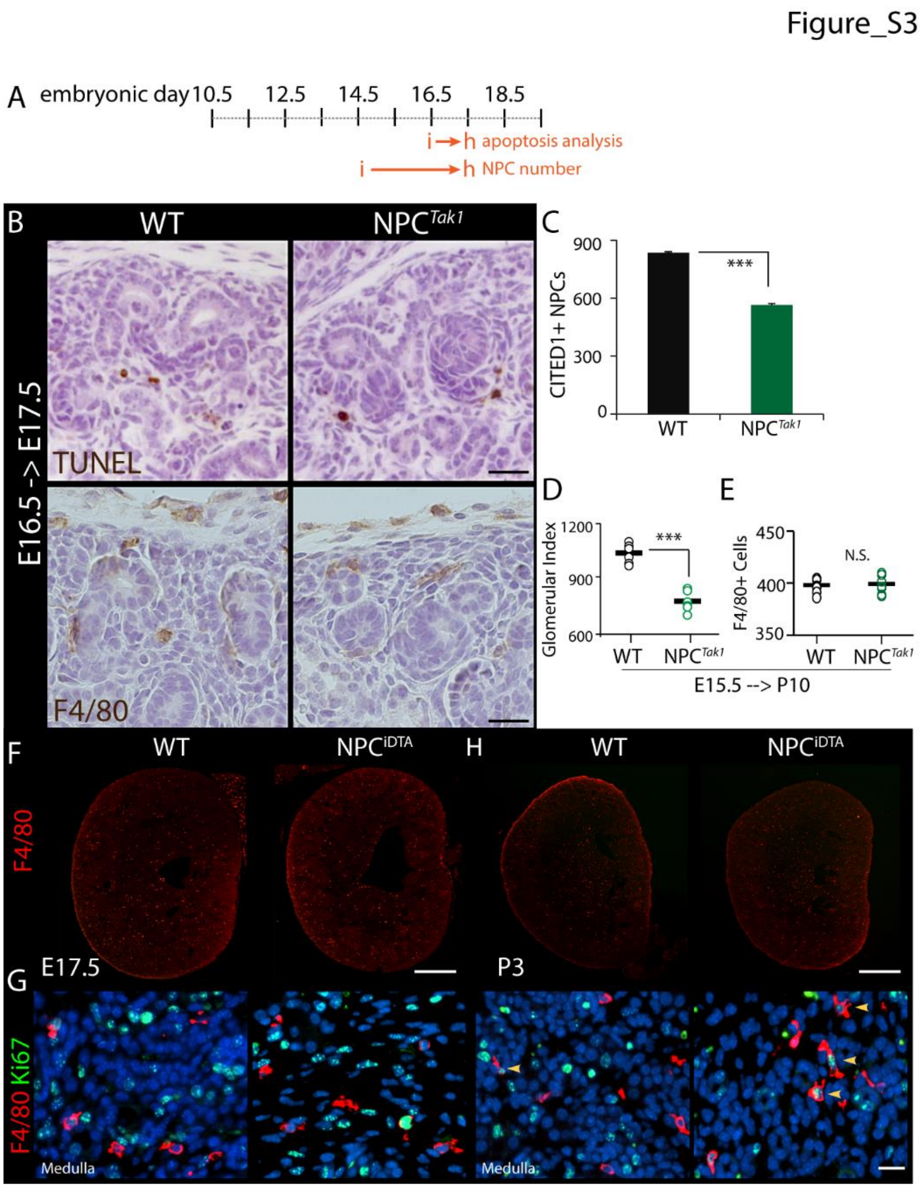

Figure S3: Comparison of NPC*Tak1* and NPCiDTA kidneys

(A) Schematic shows time points at which apoptosis and NPC cell numbers were analyzed after tamoxifen induction in WT (*Tak1*$^{c/c}$) and NPC^{Tak1} (*Cited1-creER*^{T2};*Tak1*$^{c/c}$) kidneys.

(B) TUNEL and F4/80 staining in 24 hour tamoxifen treated WT and NPC^{Tak1} kidneys.

Disease Models & Mechanisms • Supplementary information

(C) Graph shows number of CITED1+ NPCs scored per kidney section in 72 hours tamoxifen treated WT and NPC^{Tak1} kidneys. Number of individual tamoxifen treated animals analyzed per group, n=8.

(D,E) Glomerular index and number of F4/80+ cells scored per P10 WT and NPC^{Tak1} kidneys injected at E15.5. Number of individual tamoxifen treated animals analyzed per group, n=3.

(F) F4/80 (red) staining in whole kidneys from E17.5 and P3 WT and NPC^{iDTA} mice injected at E15.5.

(G) F4/80 (red) and Ki67 (green) co-staining in E17.5 and P3 WT and NPC^{iDTA} kidneys injected at E15.5. Yellow arrow heads point to F4/80+ Ki67+ cells. Data represents mean ±SD. N.S. not significant (P>0.05), **P<0.005, ***P<0.0005 Student's t-test. Scale bars, 100 µm (B) 500 µm (F) and 50 µm (G).

Figure _S4

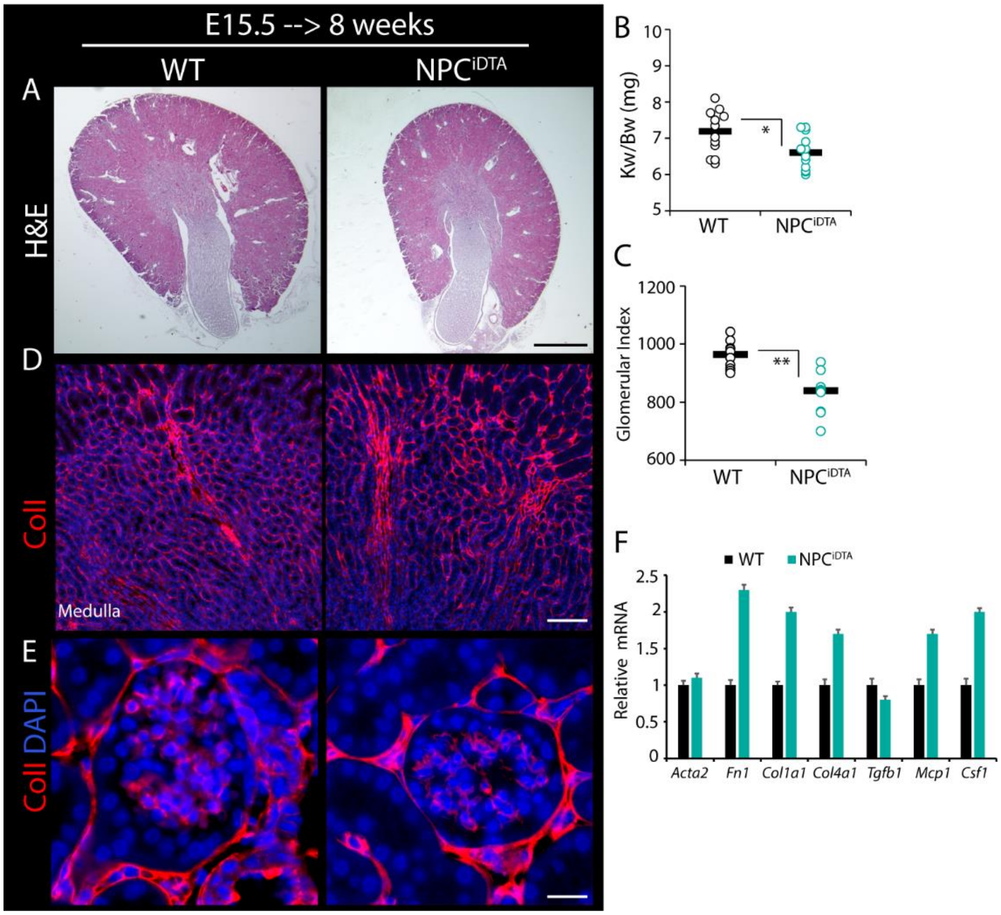

Figure S4: Analysis of 8 weeks NPC^{iDTA} kidneys

(A) H&E stained images of WT and NPC^{iDTA} kidneys tamoxifen treated at E15.5 and harvested at 8 weeks.

(B) Kidney weights normalized to body weights of WT and NPC^{iDTA} kidneys. Number of mice analyzed per group (n=6).

(C) Glomerular index of WT and NPC^{iDTA} kidneys.

(D,E) Collagen I (ColI, red) staining in medullary region and glomeruli of WT and NPC^{iDTA} kidneys.

Disease Models & Mechanisms • Supplementary information

(F) Transcriptional analysis of ECM markers and chemokines in WT and NPC^{iDTA} kidneys. Four biological replicates (n=4) analyzed per genotype. Data represents mean ±SD. N.S. not significant (*P*>0.05), **P*<0.05, ***P*<0.005 Student's t-test. Scale bars, 500 μm (A) 100 μm (D) and 50 μm (E).

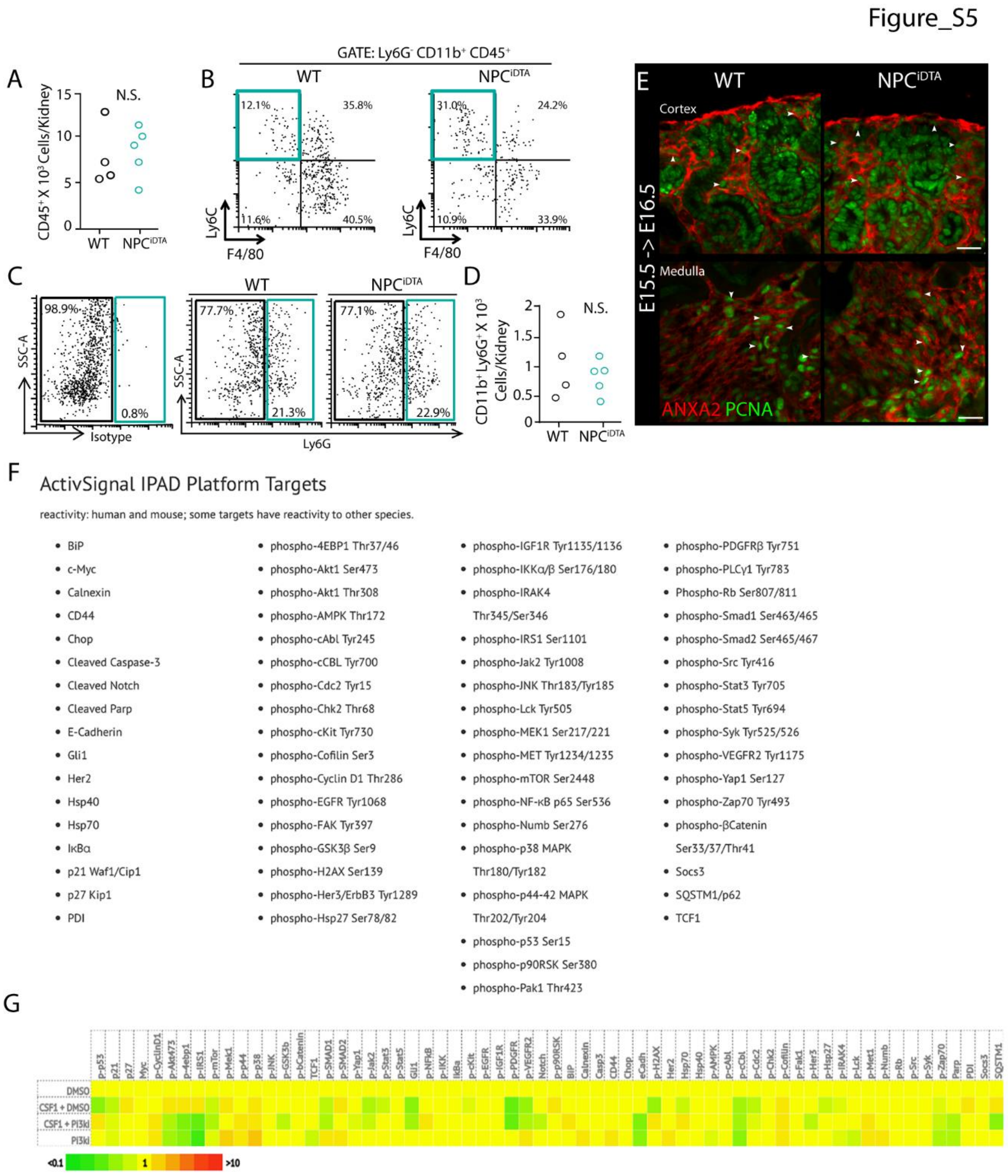

Figure S5: Functional characterization of inflammatory cells in NPC^{iDTA} kidneys

(A) Number of CD45+ cells per kidney of 24 hours tamoxifen treated E16.5 WT and NPC^{iDTA} mice.

Disease Models & Mechanisms • Supplementary information

(B) Subpopulations of myeloid mononuclear cells. $Ly6C^+$ monocytes (blue, upper left quadrant) and $Ly6C^-F4/80^+$ macrophages (lower right) within $CD11b^+CD45^+Ly6G^-$ population.

(C,D) Dot plots demonstrate percent of $Ly6G^-$ cells (black gate, myeloid mononuclear cells) and $Ly6G^+$ cells (blue gate, myeloid granulocytes) within $CD11b^+CD45^+$ cell subpopulation. Left plot - isotype-matched control antibody; middle and right plots - Ly6G antibody. Number of $Ly6G^+CD11b^+$ granulocytes scored per kidney is shown in the graph.

(E) ANXA2 (red, interstitial cell marker) and PCNA (green, proliferation marker) staining in E16.5 kidneys injected at E15.5.

(F,G) Table shows the list of ActivSignal IPAD platform targets and heatmap shows the expression profiles of all the signaling pathway components analyzed in NPCs treated with vehicle, CSF1 and PI3K inhibitor. Expression analysis was performed in two biological replicates per condition.

N.S. indicates non-significant $P>0.05$, Student's t-test. Scale bars, 100 μm.

Disease Models & Mechanisms • Supplementary information

Figure_S6

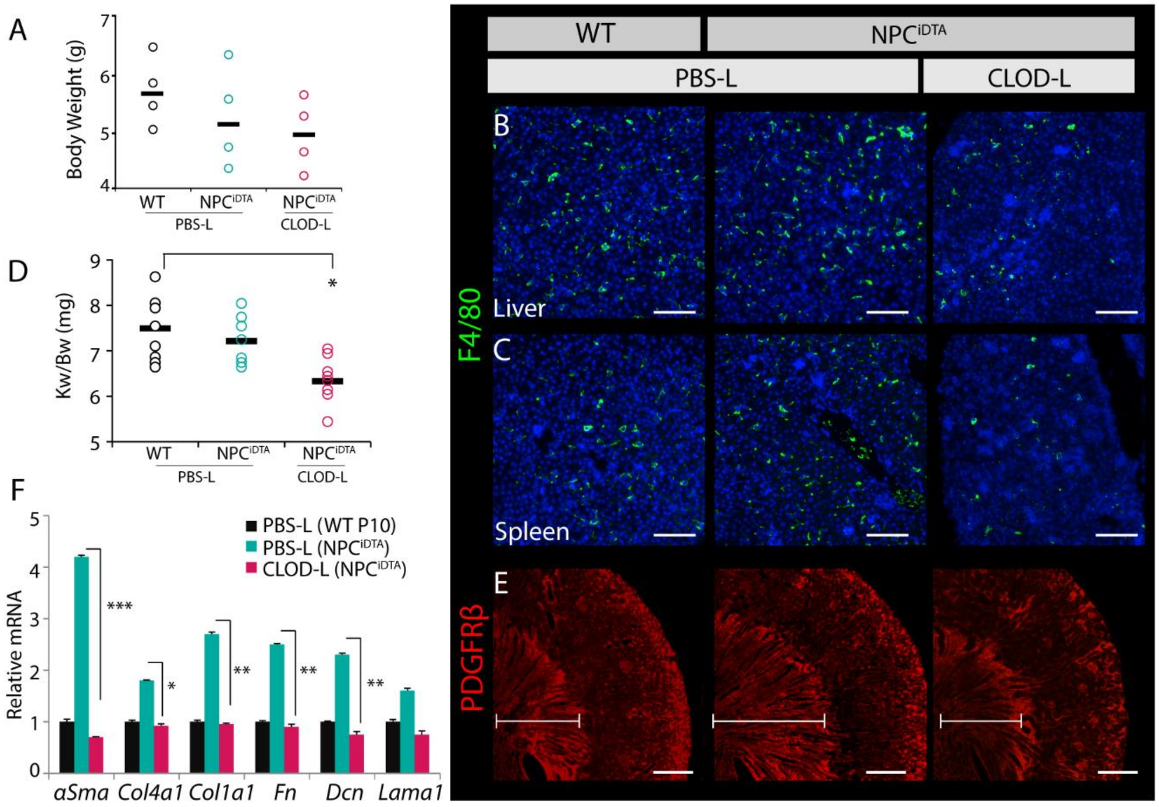

Figure S6: Clodronate mediated depletion of F4/80+ macrophages in NPC^{iDTA} kidneys

(A) Body weights of clodronate (CLOD-L) and PBS liposomes (PBS-L) treated P10 WT and NPC^{iDTA} mice.

(B,C) F4/80 (green) staining in liver and spleen

(D) Kidney weights (mg) normalized to body weights.

(E) PDGR-β staining.

(F) Transcriptional analysis of *αSma* and ECM proteins in whole kidneys. Data represents mean ±SD. N.S. not significant (P>0.05), *P<0.05, **P<0.005, and ***P<0.0005, Student's t-test. Scale bars, 100 µm in all images.

Disease Models & Mechanisms • Supplementary information

Table S1: Antibodies

	Primary antibody	Manufacturer	Dilution	Secondary antibody
1	CITED1	Neomarkers	1:200	Donkey anti-rabbit IgG Alexa Fluor 488/568
2	SIX2	Proteintech	1:250	Donkey anti-rabbit IgG Alexa Fluor 488/568
3	Cleaved Caspase 3	Cell Signaling	1:100	Donkey anti-rabbit IgG Alexa Fluor 488/568
4	pHH3	Cell Signaling	1:100	Donkey anti-mouse IgG Alexa Fluor 647/568
5	CK8/Cytokeratin-8	Developmental Studies Hybridoma Bank/DSHB	1:100	Donkey anti-rat IgG Alexa Fluor 488
6	F4/80	Santacruz Biotechnology	1:50	Donkey anti-rat IgG Alexa Fluor 488/568
7	Biotinylated-DBA Lectin	Vector Laboratories	1:100	Streptavidin- 488/568
8	GFP	Abcam	1:100	Goat anti-chicken IgY Alexa Fluor 488
9	WT1	Santacruz Biotechnology	1:100	Biotinylated anti-rabbit IgG, Streptavidin-HRP, DAB stain
10	αSMA	Sigma-Aldrich	1:100	Donkey anti-mouse IgG Alexa Fluor 568
11	PDGFRβ	Abcam	1:200	Donkey anti-rabbit IgG Alexa Fluor 568
12	Ki67	Abcam	1:100	Donkey anti-rabbit IgG Alexa Fluor 488
13	ANNEXINA2/ANXA2	Cell Signaling	1:200	Donkey anti-rabbit IgG Alexa Fluor 568
14	PCNA	Santacruz Biotechnology	1:100	Donkey anti-mouse IgG Alexa Fluor 647/568
15	Collagen I	Novus Biologicals	1:100	Donkey anti-rabbit IgG Alexa Fluor 568/488

Disease Models & Mechanisms • Supplementary information

Table S2: Mouse Primer Sequences

Gene	Primer Sequence (Forward)	Primer Sequence (Reverse)
Gapdh	AGGTCGGTGTGAACGGATTTG	TGTAGACCATGTAGTTGAGGTCA
Cd11b	CCATGACCTTCCAAGAGAATGC	ACCGGCTTGTGCTGTAGTC
Mcp1	ATCCCAATGAGTAGGCTGGAGAGC	TCAGCACAGACCTCTCTCTTGAGC
Csf1	GAACACTGTAGCCACATGATTGG	TTGACTGTCGATCAACTGCTG
aSma	GTCCCAGACATCAGGGAGTAA	TCGGATACTTCAGCGTCAGGA
Col4a1	ATGGCTTGCCTGGAGAGATAGG	TGGTTGCCCTTTGAGTCCTGGA
Col1a1	CCTCAGGGTATTGCTGGACAAC	CAGAAGGACCTTGTTTGCCAGG
Fibronectin	CCCTATCTCTGATACCGTTGTCC	TGCCGCAACTACTGTGATTCGG
Decorin	ACTCTCCAGGAACTTCGTGTCC	AGTCCCTGGAAGGCTCCGTTTT
Laminin	ATGAGCTGCAAGGAAAACTATCC	CTGTTTCGTTGGCTTCACTGA
Il6	GCAGCATCACCTTCGCTTAGA	CAGATATTGGCATGGGAGCAAG
Tgfb1	CTCCCGTGGCTTCTAGTGC	GCCTTAGTTTGGACAGGATCTG
Cxcl1	GACCATGGCTGGGATTCACCT	TCAGGGTCAAGGCAAGCCTC
Cxcl5	TGCCCTACGGTGGAAGTCATA	TGCATTCCGCTTAGCTTTCTTT
Fgf9	ACAGTGGACTCTACCTCGGCAT	GGTTGGAAGAGTAGGTGTTGTAC
Fgf20	CGGCAGGATCACAGTCTCTTCG	GTCATTCATCCCAAGGTACAGGC
Wnt9b	AGAGAGGAAGCAAGGACCTGAG	GAGAGCTGCTTCCAACAGGTA
Bmp7	TGTACGTCAGCTTCCGAGAC	GTAGTAGGCAGCATAGCCTTCA

Disease Models & Mechanisms • Supplementary information
